# Chromothripsis-Mediated Small Cell Lung Carcinoma

**DOI:** 10.1158/2159-8290.CD-24-0286

**Published:** 2024-08-26

**Authors:** Natasha Rekhtman, Sam E. Tischfield, Christopher A. Febres-Aldana, Jake June-Koo Lee, Jason C. Chang, Benjamin O. Herzberg, Pier Selenica, Hyung Jun Woo, Chad M. Vanderbilt, Soo-Ryum Yang, Fei Xu, Anita S. Bowman, Edaise M. da Silva, Anne Marie Noronha, Diana L. Mandelker, Miika Mehine, Semanti Mukherjee, Juan Blanco-Heredia, John J. Orgera, Gouri J. Nanjangud, Marina K. Baine, Rania G. Aly, Jennifer L. Sauter, William D. Travis, Omid Savari, Andre L. Moreira, Christina J. Falcon, Francis M. Bodd, Christina E. Wilson, Jacklynn V. Sienty, Parvathy Manoj, Harsha Sridhar, Lu Wang, Noura J. Choudhury, Michael Offin, Helena A. Yu, Alvaro Quintanal-Villalonga, Michael F. Berger, Marc Ladanyi, Mark T.A. Donoghue, Jorge S. Reis-Filho, Charles M. Rudin

**Affiliations:** 1 Department of Pathology and Laboratory Medicine, Memorial Sloan Kettering Cancer Center, New York, New York.; 2 Marie-Josée and Henry R. Kravis Center for Molecular Oncology, Memorial Sloan Kettering Cancer Center, New York, New York.; 3 Human Oncology and Pathogenesis Program, Memorial Sloan Kettering Cancer Center, New York, New York.; 4 Department of Medicine, Memorial Sloan Kettering Cancer Center, New York, New York.; 5 Division of Hematology and Oncology, Department of Medicine, Columbia University Irving Medical Center and the Herbert Irving Comprehensive Cancer Center, New York, New York.; 6 Department of Molecular Cytogenetics Core Facility, Memorial Sloan Kettering Cancer Center, New York, New York.; 7 Department of Pathology, University Hospitals Cleveland Medical Center- Case Western Reserve University, Cleveland, Ohio.; 8 Department of Pathology, New York University Grossman School of Medicine, New York, New York.; 9 Druckenmiller Center for Lung Cancer Research, Memorial Sloan Kettering Cancer Center, New York, New York.; 10 Division of Biostatistics Research Scientists, New York University, New York, New York.; 11 Department of Pathology, St. Jude Children’s Research Hospital, Memphis, Tennessee.; 12 Department of Medicine, Weill Cornell Medical College, New York, New York.

## Abstract

Small cell lung carcinoma (SCLC) is a highly aggressive malignancy that is typically associated with tobacco exposure and inactivation of *RB1* and *TP53* genes. Here, we performed detailed clinicopathologic, genomic, and transcriptomic profiling of an atypical subset of SCLC that lacked *RB1* and *TP53* co-inactivation and arose in never/light smokers. We found that most cases were associated with chromothripsis—massive, localized chromosome shattering—recurrently involving chromosome 11 or 12 and resulting in extrachromosomal amplification of *CCND1* or co-amplification of *CCND2*/*CDK4*/*MDM2*, respectively. Uniquely, these clinically aggressive tumors exhibited genomic and pathologic links to pulmonary carcinoids, suggesting a previously uncharacterized mode of SCLC pathogenesis via transformation from lower-grade neuroendocrine tumors or their progenitors. Conversely, SCLC in never-smokers harboring inactivated *RB1* and *TP53* exhibited hallmarks of adenocarcinoma-to-SCLC derivation, supporting two distinct pathways of plasticity-mediated pathogenesis of SCLC in never-smokers.

**Significance:** Here, we provide the first detailed description of a unique SCLC subset lacking *RB1*/*TP53* alterations and identify extensive chromothripsis and pathogenetic links to pulmonary carcinoids as its hallmark features. This work defines atypical SCLC as a novel entity among lung cancers, highlighting its exceptional histogenesis, clinicopathologic characteristics, and therapeutic vulnerabilities.

*See related commentary by Nadeem and Drapkin, p. 8*

## Teaser

This study describes an exceptional, alternative pathway of SCLC pathogenesis via chromothripsis and ecDNA in the absence of *RB1* and *TP53* mutations or smoking mutagenesis, defining a novel tumor type associated with distinct prognosis and therapeutic profile.

## Introduction

Small cell lung carcinoma (SCLC) is an extremely aggressive malignancy, characterized by a nearly universal genomic inactivation of *RB1* and *TP53*, a high tumor mutational burden (TMB), and nearly invariable association with cigarette smoking (1). We recently described an uncommon subset of SCLC with *RB1* proficiency, characterized by expression of wild-type *RB1* in tumors that otherwise exhibited classic properties of SCLC, including *TP53* mutations, high TMB, and history of smoking. Conversely, the characteristics of the exceptional cases of SCLC lacking both of its hallmark genomic alterations—*RB1* and *TP53*—remain almost entirely undefined.

In a prior whole-genome sequencing (WGS) study of 110 SCLC cases, only two cases were identified that harbored wild-type *RB1* and *TP53* (2). Although not the focus of that prior report, it is notable that both cases demonstrated evidence of chromothripsis—a process of localized massive chromosome shattering— involving chromosomes 11 and 3, and associated with overexpression of *CCND1* on chromosome 11. This suggested that the pathogenesis of SCLC with intact *RB1* and *TP53* may be mediated by an entirely distinct mechanism; however, further details on clinicopathologic characteristics of such tumors and analysis of this phenomenon at scale have been lacking. Furthermore, in a cohort of 3,600 SCLC samples submitted for broad targeted next-generation sequencing (tNGS) at Foundation Medicine, 5.5% were reported to lack *RB1* and *TP53* alterations; this subset harbored instances of *CCND1* and *MDM2* amplification, but no WGS or detailed clinicopathologic characterization of these samples was available (3). Furthermore, as recently demonstrated, tNGS may miss deleterious *RB1* alterations in a substantial proportion of cases; therefore, combining tNGS with expression data is essential for establishing *RB1* proficiency (4).

Through enterprise-wide clinical application of broad tNGS using Memorial Sloan Kettering Integrated Mutation Profiling of Actionable Cancer Targets (MSK-IMPACT; refs. 5, 6), integrated with expression-based analysis, we identified 20 patients with SCLC whose tumors lacked *RB1* and *TP53* co-inactivation. Remarkably, all identified patients were never or light smokers, defined as the lifetime smoking history of ≤10 pack-years, further highlighting the unique pathogenesis underlying these tumors. Based on prior studies, it is known that SCLC can rarely arise in never or light smokers via histologic transformation of lung adenocarcinoma (LUAD) either after treatment with targeted therapies or *de novo* (7, 8), and that *RB1* and *TP53* co-deficiency is a prerequisite for this conversion (9, 10). Conversely, SCLC in never/light smokers lacking *RB1* and *TP53* co-deficiency is a highly unusual and previously uncharacterized subset.

To better understand the pathogenetic mechanisms and clinicopathologic characteristics of this cohort, we performed a multifaceted analysis of clinical samples, which included WGS and RNA sequencing (RNA-seq) when sufficient tissue was available after tNGS. Here, we demonstrate that nearly all *RB1*/*TP53*-proficient SCLC are characterized by extensive chromothripsis, associated with extrachromosomal (ecDNA) amplification of *CCND1*, *CCND2*, *CDK4*, and *MDM2*. Furthermore, we present evidence for a histogenetic relationship of these tumors with a separate class of neuroendocrine cancers–pulmonary carcinoids.

Pulmonary carcinoids, alternatively termed neuroendocrine tumors (NET), are regarded as an entirely separate class of tumors from SCLC. These are generally indolent neuroendocrine neoplasms, which lack an association with tobacco exposure and arise in younger patients than SCLC (11, 12). They are characterized genomically by a low TMB, recurrent alterations in *MEN1*, *EIF1AX*, and *ARID1A*, and, notably, the lack of *RB1* and *TP53* alterations (12, 13). Pathologically, pulmonary carcinoids exhibit minimal to intermediate proliferation rate, featuring Ki67 index of 1% to 30% (12). Although proliferative escalation has been documented in metastasizing carcinoids (14), whether carcinoids can exhibit a full phenotypic conversion to SCLC has remained a controversial concept, lacking robust clinicopathologic and molecular documentation. The data presented here suggest a new mode of SCLC pathogenesis through a histogenetic link to carcinoids or their progenitors through chromothripsis-mediated cancer gene deregulation in the context of *RB1*/*TP53* proficiency.

## Results

### Analysis of *RB1*, *TP53*, and Smoking Status to Identify “Atypical SCLC”

**Figure 1.**
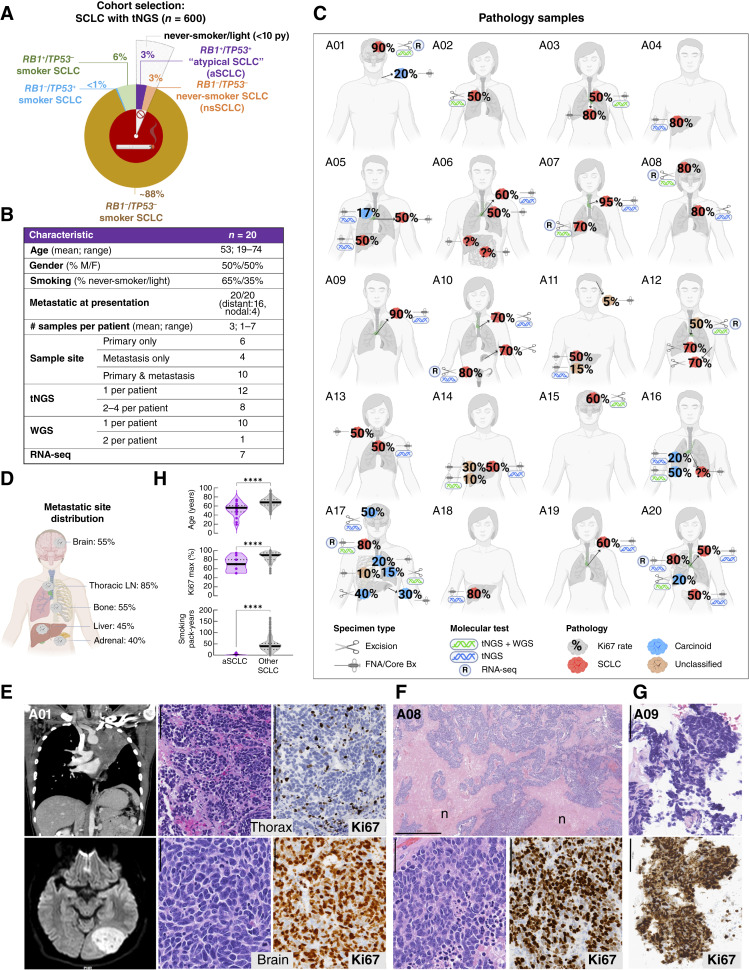
Cohort selection and clinicopathologic characteristics. **A,** Schematic diagram of *RB1* and *TP53* inactivation (outer doughnut) and smoking status (inner doughnut) in a cohort of 600 consecutive SCLC that underwent tNGS by MSK-IMPACT. *RB1* and *TP53* status was determined based on integrated genomic and IHC data (see “Results”). **B,** Tabular and (**C**) pictorial summary of pathologic sample characteristics and sequencing assays performed for 20 patients with aSCLC. See “Methods” for histopathologic criteria. Ki67 not available. **D,** Summary of metastatic site distribution based on radiologic–pathologic findings (full data in Supplementary Table S2). **E–G,** Compilation of radiologic and pathologic findings for three representative patients. **E,** (A01): 19-year-old patient with brain metastasis showing classic undifferentiated morphology of SCLC: small, crowded/molding cells, numerous mitotic figures (>50/2 mm^2^), apoptotic bodies, and Ki67 of 90%, whereas thoracic sample shows well-differentiated, nested morphology of a carcinoid tumor lacking mitotic figures or apoptotic bodies, and exhibiting Ki67 of 5%–20%. **F,** Case A08 and (**G**) case A09 illustrate classic SCLC morphology in primary lung tumor samples, featuring Ki67 rate of 80% and 90%, respectively. *n* indicates areas of extensive necrosis–a hallmark feature of SCLC. For extended pathologic illustrations, see Supplementary Figs. S1–S4. Scale bars are 100 μm for Ki67 and 50 μm for H&E except for top in **F** which is 1 mm. **H,** Comparison of age, Ki67 proliferation index, and smoking pack-years in aSCLC vs. other SCLC (*n* = 224 for age and Ki67; *n* = 200 for pack-year smoking). For patients with multiple samples, Ki67 represents the maximal (max) hot-spot rate among all evaluated samples. H&E, hematoxylin and eosin; FNA, fine needle aspiration; Core bx, core biopsy; py, pack-years; tNGS, targeted next-generation sequencing. (**C, D** created in part with BioRender.com.)

As depicted in Fig. 1A, among the first 600 patients diagnosed with *de novo* SCLC who underwent sequencing by MSK-IMPACT, 20 (3%) exhibited *RB1* and *TP53* dual proficiency, defined by an integrated genomic and IHC approach (Supplementary Table S1). *RB1*-proficient (*RB1*^+^) tumors lacked *RB1* genomic alterations and retained pRb protein expression by IHC, although, as discussed later, few cases exhibited subclonal or acquired *RB1* mutations in subsequent/metastatic samples. As additional support for *RB1* proficiency in initial samples, all cases had D-type cyclin^high^ and/or p16^low^ profiles, which in prior studies was exclusive to *RB1*^+^ SCLC (4). *TP53* proficiency (*TP53*^+^) was defined by a wild-type p53 pattern by IHC and the lack of *TP53* genomic alterations (see “Methods”). Remarkably, all patients with *RB1*^+^/*TP53*^+^ SCLC were never or light (<10 pack-year) smokers. The rest of the SCLC cohort was composed predominantly of smokers with *RB1*^−^/*TP53*^−^ SCLC (88%), smokers with the recently described *RB1*-proficient SCLC (*RB1*^+^/*TP53*^−^; 6%; ref. 4), and never-smokers with *RB1*^−^/*TP53*^−^ SCLC (3%; described later). Given the lack of *RB1* and *TP53* alterations, and the absent or low tobacco exposure, we designated this unique subset as atypical SCLC (aSCLC).

### Clinicopathologic Characteristics of aSCLC

The demographic and pathologic sample characteristics of patients with aSCLC are summarized in Fig. 1B and C and Supplementary Tables S2 and S3. Patients had 1 to 7 pathologic specimens obtained during the course of disease (total 49), all of which underwent detailed pathologic review, 31 (at least 1 per patient) were profiled by tNGS, 12 (from 11 patients) by WGS, and 7 by RNA-seq.

Patients presented with lung tumors measuring up to 7.2 cm (mean 3.3 cm). All patients had pathologically confirmed metastatic disease at presentation, including distant metastases in 16 patients and metastasis limited to regional lymph nodes in four patients. The most prevalent sites of distant metastases included brain (55%), bone (55%), liver (45%), and adrenal (40%)—the sites commonly involved by neuroendocrine lung cancers (Fig. 1D; Supplementary Table S2).

Pathologically, aSCLC samples exhibited classic SCLC morphology, manifesting as primitive, crowded cells with a high nuclear-to-cytoplasmic ratio, extensive necrosis, and a high Ki67 proliferation index (in most cases 70%–>90%; Fig. 1E–G). All aSCLC expressed multiple neuroendocrine markers by IHC, including synaptophysin, chromogranin A, CD56/NCAM, and INSM1 (full IHC results are summarized in Supplementary Table S2, and illustrated in Supplementary Figs. S1–S3, S4A, and S4B). Uniquely, in five patients (cases A02, A05, A16, A17, and A20), the SCLC histotype was present in metastatic sample(s), whereas primary/intrathoracic sample(s) exhibited a carcinoid histotype, characterized by well-differentiated bland, uniform cells with a lower nuclear-to-cytoplasmic ratio, and Ki67 index of mostly ≤20% (Fig. 1E; Supplementary Figs. S3 and S4A).

Compared with other SCLC, patients with aSCLC exhibited several distinct clinicopathologic characteristics (Fig. 1H). First, patients with aSCLC were significantly younger, as young as 19 years at presentation (mean age 53 vs. 67 years, respectively; *P* < 0.0001). Second, although Ki67 proliferation index in all aSCLC samples was in the range of conventional SCLC (50%–100%), these tumors were enriched in Ki67 rates at the lower end of this spectrum compared with other SCLC (mean Ki67 rate 69% vs. 87%, respectively; *P* < 0.0001). Lastly, the absent/low pack-year smoking history in aSCLC contrasted sharply with pack-year smoking in other SCLC (*P* < 0.0001). Full comparison of the demographic and clinicopathologic characteristics of aSCLC versus other SCLC is shown in Supplementary Table S4.

### Genomic Profiling of aSCLC Reveals Low TMB and Recurrent Oncogene Amplifications

**Figure 2.**
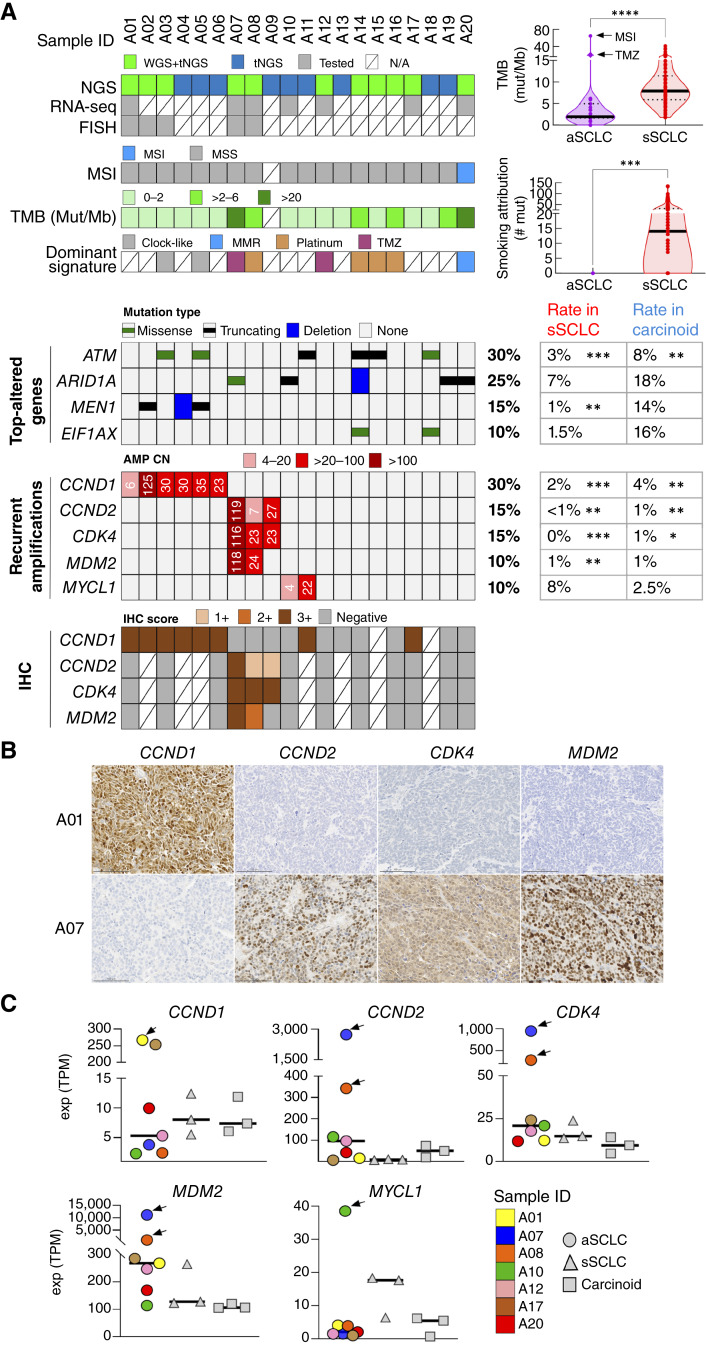
Mutation and CNA landscape of aSCLC. **A,** Mutations and CNAs in aSCLC in comparison to the control groups of sSCLC (*n* = 206) and pulmonary carcinoids (*n* = 157) analyzed by MSK-IMPACT. OncoPrint summarizing assays performed and overall genomic features: MSI vs. MSS, TMB, and genomic signature (full signature analysis shown in Supplementary Fig. S5). Case A09 lacked a matched normal DNA sample and was therefore excluded from TMB, MSI, and signature analysis. Displayed genomic alterations include selected recurrently altered genes (see Supplementary Tables S5 and S6 for a full list of detected alterations). For patients with multiple samples, the index sample is displayed (see “Methods”). *, *P* < 0.05; **, *P* < 0.01; ***, *P* < 0.001; ****, *P* < 0.0001. **B,** Expression of recurrently amplified genes by IHC in two representative cases (scale bars, 100 μm) and (**C**) by RNA-seq in cases with available data. Arrows in **C** indicate cases harboring corresponding amplifications. TPM, transcripts per million; MMR, mismatch repair; N/A, not available; MSS, microsatellite stable.

The overall mutational landscape in aSCLC was initially assessed using the MSK-IMPACT assay for all 20 patients, which was performed with a mean coverage depth of 613× (range, 255–959×; Supplementary Table S5). The results were compared with those in smoking-associated SCLC (sSCLC; *n* = 206) and pulmonary carcinoids (*n* = 157) analyzed on the same platform (Fig. 2A).

TMB in aSCLC was remarkably low, with most cases exhibiting TMB of <2 mutations per Mb; two outliers featuring high TMB were from a sample obtained after temozolomide (TMZ) treatment (A07) and a unique case in this study exhibiting microsatellite instability (MSI; case A20). Tumors with a sufficient number of single-nucleotide variants (SNV) to assess mutational signature consistently lacked a smoking signature and primarily exhibited a dominant signature reflecting systemic therapy received (Supplementary Fig. S5A–S5C). The low TMB and absence of a smoking signature in aSCLC contrasted sharply with the findings in sSCLC (Fig. 2A).

Recurrent genomic alterations in aSCLC were dominated by mutations characteristic of lung carcinoids (*MEN1*, *EIF1AX*, *ARID1A*, and *ATM*), with an enrichment in the rate of *ATM* mutations (30%) compared with both carcinoids (8%; *P =* 0.008) and SCLC (3%; *P =* 0.0003; Fig. 2A; see Supplementary Tables S5 and S6 for full SNV data).

A striking feature of aSCLC was the presence of recurrent and mutually exclusive amplifications of several key oncogenes controlling cell cycle and survival: *CCND1* (30%), *CCND2*/*CDK4*/±*MDM2* (15%), and *MYCL1* [10%; Fig. 2A; full copy number alteration (CNA) data are provided in Supplementary Tables S5 and S7]. These amplifications were mostly present at a high copy number (CN; >10), and some exceeded 100 copies (such as case A02, harboring 125 copies of *CCND1*). Conversely, amplifications of these genes were seen only rarely in conventional SCLC and carcinoids; for example, *CDK4* amplifications were found in 0% of SCLC (*P* = 0.0006) and 1% of carcinoids (*P* = 0.01). Amplifications were consistently accompanied by overexpression of the corresponding proteins by IHC (Fig. 2B) and mRNA (Fig. 2C). Of note, two cases (A11 and A17) showed *CCND1* overexpression by mRNA and IHC in the absence of *CCND1* amplification at the genomic level, indicating that *CCND1* overexpression may be mediated by other mechanisms in some aSCLC, as discussed later.

The sole MSI-high (MSI-H) case (A20) was characterized by somatic *MLH1* homozygous deletion and dual loss of MLH1/PMS2 by IHC. Using the same methods, the MSI rate in conventional lung carcinomas was restricted to rare cases in a recent series from our institution [1.9% for SCLC and 0.4% for non-SCLC (NSCLC; ref. 15); in the control set of lung carcinoids (*n* = 157), none were found to be MSI-H. Pathologically, A20 was one of the cases with SCLC histotype in metastatic samples (Ki67 50%–80%) and carcinoid histotype in the primary tumor (Ki67 5%–20%; Supplementary Fig. S4).

### Chromothripsis as the Underlying Mechanism for Amplifications

**Figure 3.**
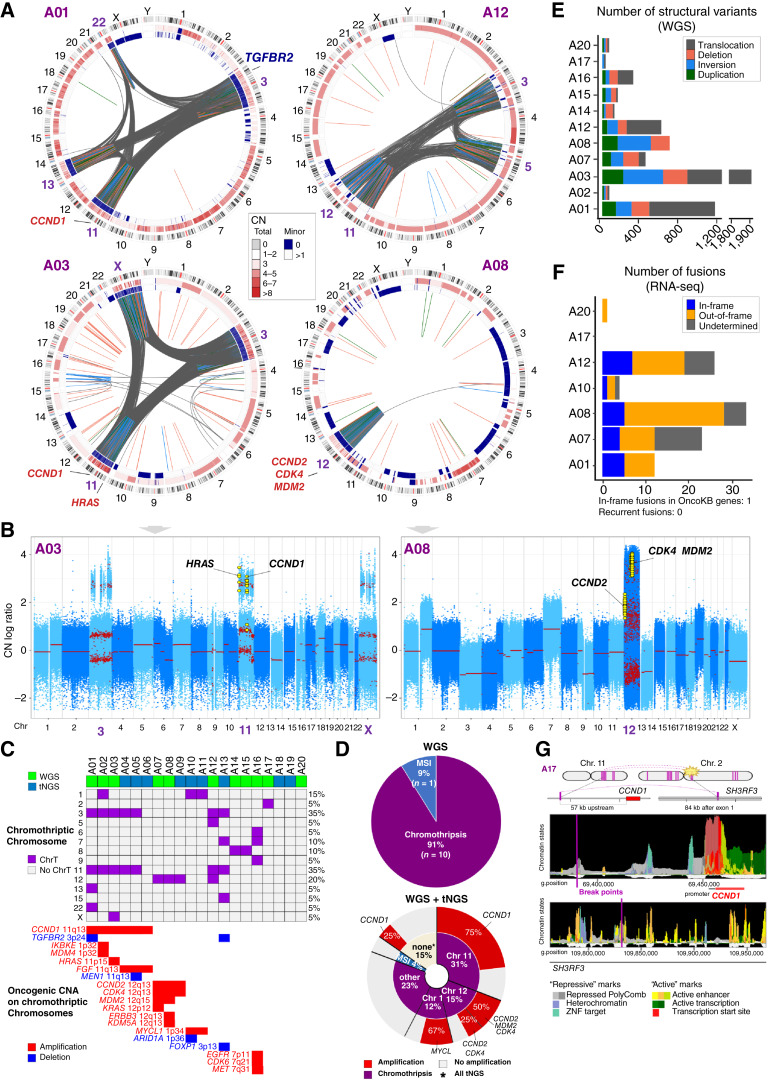
Chromothripsis and corresponding cancer gene amplifications and losses. **A,** Representative Circos plots of SVs and CNAs across the genome by WGS. Outer band shows an ideogram of chromosome positions and cytogenetic bands. Second band depicts total CN, and third band shows minor allele CN. The inner circle depicts SVs as arcs connecting the two relevant genomic points as identified by three algorithms (see “Methods”). CNAs in key cancer genes in the regions of chromothripsis (red, amplifications; blue, deletions) are displayed. Circos plots for all cases are shown in Supplementary Fig. S6. **B,** CN log ratio plots from the FACETS algorithm displaying the distinctive oscillating CN states on chromosomes with chromothripsis. CN segments are shown in red. Focal segments (<2 MB in size) are shown as enlarged points for visual purposes. Selected amplifications are indicated (yellow). **C,** Summary of chromosomal location of chromothriptic events in cases analyzed by WGS and tNGS. Also shown are selected amplifications and losses in oncogenes and tumor suppressors, respectively, localized to the chromothriptic chromosomes. Full list is provided in Supplementary Table S7. **D,** Schematic summary for the rate of major genomic mechanisms detected in the set analyzed by WGS (*n* = 11) and in the full cohort (*n* = 20). In the lower diagram, major chromosomes involved by chromothripsis are indicated in the inner doughnut, and corresponding recurrent gene amplifications are indicated in the outer doughnut. **E,** Total number of SVs identified in samples analyzed by WGS. Variants are color-coded by type. **F,** Number of fusions predicted in samples with available RNA-seq. **G,** Diagram illustrating putative enhancer hijacking in case A17 with chromothripsis on chromosome 2 resulting in translocation between *SH3RF3* on chromosome 2 and upstream regulatory region of *CCND1* on chromosome 11. Epigenetic landscape surrounding the breakpoint was extrapolated from data from multiple tissue types (Epilogos search tool). ChrT, chromothripsis.

To elucidate the underlying genomic processes leading to aSCLC, samples from 11 patients with sufficient residual DNA after MSK-IMPACT were further analyzed by WGS. This confirmed the lack of pathogenic *RB1* and *TP53* alterations in all tested samples, except for an unusual subclonal *RB1* mutation in A12, discussed later (Supplementary Table S8). Remarkably, all but one case (MSI-H, A20) revealed the hallmark features of chromothripsis, as evidenced by clustered massive structural variants (SV; Fig. 3A and B; Supplementary Fig. S6) associated with co-localized CNAs (summarized in Fig. 3C and D). Within the regions affected by chromothripsis, CNAs exhibited the characteristic oscillating pattern featuring alternating retained and lost genomic material, reflecting the loss of DNA segments during re-ligation of the shattered chromosomes (16). The average number of SVs per case was 565, extending to >1,900 (Fig. 3E; see Supplementary Table S9 for the full list of SVs).

Next, using cases with matched WGS and tNGS, we developed an approach for the manual detection of chromothripsis in tNGS based on the distinctive oscillating CNA pattern, which showed 100% specificity and 77% sensitivity of tNGS for the detection of chromothripsis using WGS as a gold standard (Supplementary Fig. S7A–S7C). Using this approach, we identified chromothripsis in six of nine additional cases analyzed by tNGS only. Overall, for the combined WGS and tNGS analyses, evidence of chromothripsis was identified in 16 of 19 (84%) non-MSI aSCLC.

Across cancer types, chromothripsis typically localizes to one or a few chromosomes (17, 18). Indeed, in aSCLC, on average, two chromosomes were affected per case (range, 1–4; Fig. 3C). Chromosome 11 was the most commonly affected (35% of cases), which was invariably accompanied by chromothripsis on chromosome 3. This was followed by recurrent chromothripsis on chromosome 12 (20%) and chromosome 1 (15%).

Notably, chromothripsis of specific chromosomes correlated with the amplifications of genes localized to those chromosomes. Namely, chromothripsis on chromosome 11 was observed in five of six cases with *CCND1* (11q13) amplification, chromothripsis on chromosome 12 was seen in three of three cases with *CCND2* (12q13)/*CDK4* (12q14)/±*MDM2* (12q15) co-amplification, and chromothripsis on chromosome 1 was seen in two of two cases with *MYCL1* (1p34) amplification. Of note, the sole case with *CCND1* amplification (23 copies) in the absence of detectable chromothripsis (A06) was analyzed by tNGS only; therefore, a false-negative result cannot be excluded given the incomplete sensitivity of this assay for detecting chromothripsis.

Interestingly, in two cases, chromothripsis on chromosome 11 (A12 and A13) and chromosome 12 (A12) lacked the amplifications of *CCND1* or *CCND2*/*CDK4*/*MDM2*, respectively. Thus, chromothripsis on individual chromosomes is strongly but not invariably associated with the amplification of specific oncogenes.

Beyond the recurrent amplification of *CCND1*, *CCND2*, *CDK4*, *MDM2*, and *MYCL1*, chromothriptic chromosomes were associated with the amplification or loss of other well-established cancer genes. This included *HRAS* (11p15) amplification on chromothriptic chromosome 11, *KRAS* (12q12) and *ERBB3* (12q13) amplification on chromosome 12, as well as deletions of key carcinoid-associated tumor suppressor genes *MEN1* (11q13) and *ARID1A* (1p36) on chromosomes 11 and 1, respectively. These CNAs were consistently associated with the overexpression or loss of expression of the corresponding mRNA by RNA-seq (Supplementary Fig. S8). On average, three (up to eight) genes regarded as oncogenic or likely oncogenic by the OncoKB annotation (19) exhibited amplifications or (in the case of tumor suppressors) losses on chromothriptic chromosomes (full list included in Supplementary Table S7). Overall, of the 16 cases with chromothripsis, 12 harbored oncogenic CNAs in the regions of chromothripsis.

Although each instance of chromothripsis on chromosome 11 was accompanied by chromothripsis on chromosome 3 (7/7 cases), the potential target gene(s) on chromosome 3 are unclear. The only recurrent event on chromothriptic chromosome 3 was the deletion of *TGFBR2* (3p24) in two cases; suppression of this gene has been implicated in SCLC progression (Fig. 3C; Supplementary Fig. S8; ref. 20). Loss of the 3p chromosome arm is a well-known recurrent event in SCLC, occurring in >90% of SCLC cases (21). Although the relevant target gene(s) on 3p are not well established, *ROBO1* and *FHIT5* have been suggested as potential candidates (2); no deletions (Supplementary Table S7) or loss of expression by RNA-seq were identified in these genes in our cases.

To directly compare the prevalence and extent of chromothripsis in aSCLC versus other major lung cancer types, we analyzed publicly available WGS datasets for LUAD (18) and *RB1*^−^/*TP53*^−^ SCLC (2), using similar computational methods as applied to aSCLC. We found that, as previously reported for LUAD (18), chromothripsis was present in a subset of cases; however, for both LUAD and *RB1*^−^/*TP53*^−^ SCLC, its prevalence and extent, as reflected by the number of SVs per chromothriptic chromosomes, were significantly lower than in aSCLC (Supplementary Fig. S9A and S9B).

These findings establish aSCLC as a tumor that is predominantly characterized by extensive chromothripsis, leading to amplification of oncogenes and loss of tumor suppressors, some of which have established critical roles in the biology of lung neuroendocrine cancers.

### Analysis of Oncogenic Fusions

In addition to CNAs, the other major potential functional consequence of chromothripsis is the formation of oncogenic fusions (22–24). Therefore, we investigated whether a massive number of SVs in aSCLC was associated with the formation of in-frame fusions. For cases with available RNA-seq data, we found that, despite the massive number of rearrangements by WGS, cases with chromothripsis harbored on average only 16 fusions per case (range, 0–29), of which only a small fraction (mean 3 per case, range, 0–5) were in-frame (Fig. 3F). All in-frame fusions involved genes with unknown oncogenic function by OncoKB, except for a single fusion involving *ALDH1L2*—a folate regulatory enzyme considered likely oncogenic, but with no established role in lung cancer or neuroendocrine cancers (25). Furthermore, none of the fusions were recurrent (Supplementary Table S10). We therefore conclude that, although fusions could have a contributory role, they are unlikely to be the dominant oncogenic drivers in aSCLC.

### *CCND1* Upregulation due to a Genomic Rearrangement Resulting in Enhancer Hijacking

We further asked whether chromothripsis-associated SVs could deregulate cancer genes by disrupting their regulatory sequences. In case A17, harboring chromothripsis on chromosome 2 but without associated oncogene amplifications, there was a genomic rearrangement between *SH3RF3* on chromosome 2 and a region upstream of *CCND1*. *SH3RF3* is a highly expressed gene in lung tissue [ID: ENSG00000172985 in the GTEx RNA dataset (26)], and the rearrangement juxtaposed its active chromatin marks into repressed region upstream of *CCND1*, based on the data extrapolated from multiple tissue types [Epilogos search tool (27)], suggesting a putative enhancer hijacking mechanism to upregulate *CCND1* in the absence of amplification (Fig. 3G). Indeed, marked overexpression of cyclin D1 was detected in this case by both IHC and RNA-seq (Fig. 2C). This illustrates an alternative mechanism of oncogene upregulation in aSCLC, in line with prior cancer-wide evidence for SVs involving upstream regulatory sequences representing an efficient mechanism of oncogene activation (28, 29).

### Chromothripsis through Micronucleation and Formation of ecDNA

**Figure 4.**
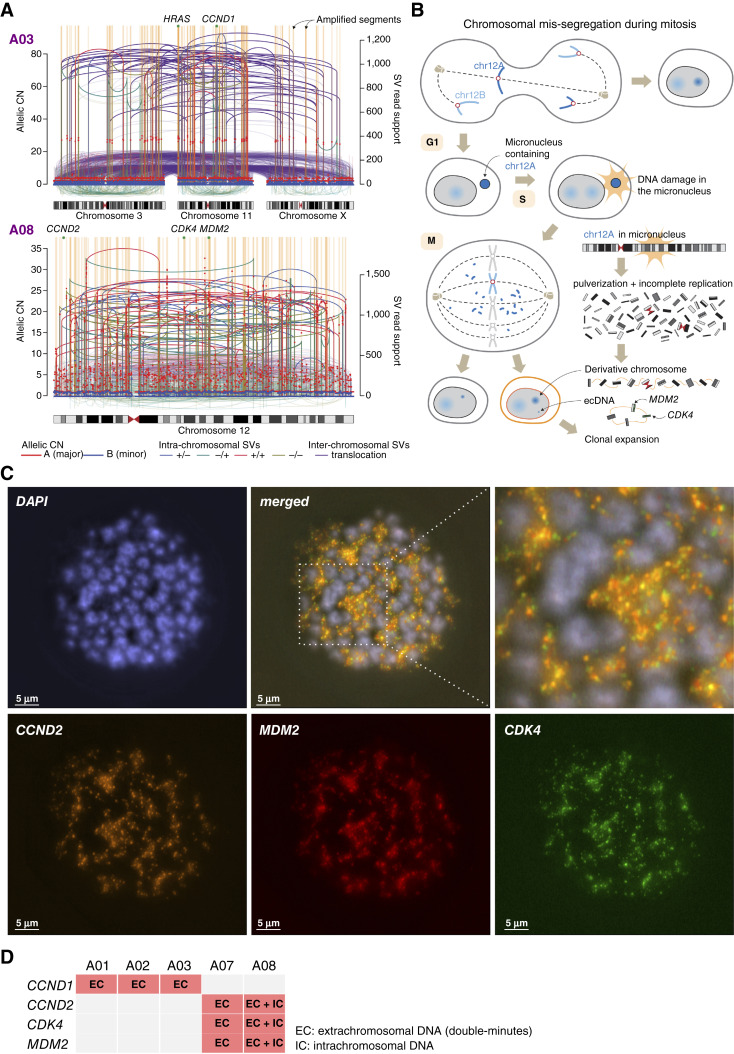
Chromosomal architecture of chromothripsis and amplifications. **A,** Integrated SV and CNA analysis in two representative cases confirming the hallmark features of chromothripsis and suggesting formation of ecDNA and micronucleation as a mechanism of gene amplification and chromothripsis (see “Results”). Allele-specific CN for each genomic segment is shown on the left *y*-axis. Genomic segments in which the CN is greater than 5 times of chromosomal baseline are indicated as orange shades to highlight the most significant amplification events. Vertical lines and arcs indicate genomic breakpoints and connections of SVs, of which the number of supporting read fragments is shown on the right *y*-axis. Colors of the SVs are based on the orientation of paired reads. All cases are shown in Supplementary Fig. S10. **B,** Conceptual diagram explaining the mechanism of chromothripsis and oncogene amplification in aSCLC. **C,** FISH analysis of case A07 with 3-color probes: *MDM2* (red), *CDK4* (green), and *CCND2* (orange), revealing ecDNA location of amplified genes (so-called “double-minutes”). Sample is of a metaphase spread from a patient-derived xenograft (see “Methods”). **D,** Summary of FISH results for all tested samples.

Next, we explored the architecture of oncogene amplification and potential generative mechanisms of chromothripsis by integrative analysis of SV breakpoints and associated CNAs in WGS. This confirmed the cardinal features of chromothripsis, including characteristic CN oscillations and numerous SVs interleaved in random orientations, indicating ligations between DNA fragments in a random manner (Fig. 4A). Previous studies reported that chromothripsis can be observed either in a whole chromosome or more locally (18); in seven of our cases, the chromothripsis events involved the entire length of the affected chromosomes (Supplementary Fig. S10). Whole-chromosome involvement with evidence of heavy fragmentation and re-ligation suggests that chromosomal mis-segregation during mitosis, micronucleation, and subsequent massive DNA damage and rearrangements represent an underlying mechanism of chromothripsis in aSCLC (Fig. 4B; refs. 16, 30).

In addition, a group of genomic segments exhibited far higher CNs than the other chromothriptic segments, while maintaining other features of chromothripsis. This suggested the direct formation of circular ecDNA from chromothripsis rather than amplicon formation subsequent to chromothripsis (31). Some oncogenes were further amplified up to twice the CN level of the other ecDNA fragments [e.g., *CCND2* in A08; Fig. 4A (bottom)], indicating early duplication of the oncogenes within the ecDNA and subsequent high-level amplification.

To further corroborate the nature of amplification, we performed FISH for *CCND1* or *CCND2*/*CDK4*/*MDM2* in cases with corresponding amplifications and sufficient residual tissue for this analysis (*n* = 5). In all cases, this confirmed ecDNA (“double-minute”) amplification of the tested genes, with one case exhibiting both extra- and intrachromosomal amplifications (Fig. 4C and D).

### Analysis of Potential Predispositions to Chromothripsis in aSCLC, Including Germline Analysis

We next sought to examine whether patients with aSCLC harbored potential predispositions that were previously implicated as risk factors for chromothripsis.

Given the previously described association of chromothripsis with germline mutations in *ATM* and *TP53* (32–34), we reviewed the germline data for the aSCLC cohort. Germline variant calls were evaluable for 17 of the 20 patients and revealed no pathogenic germline variants in any of the 90 tested genes associated with hereditary cancer susceptibility. Although no germline *ATM* mutations were identified, the enrichment in somatic *ATM* mutations may be of interest. ATM blocks cell-cycle progression in the presence of DNA double-strand breaks (35), and disabling this checkpoint could provide a permissive environment for the development of aSCLC. In model systems, ATM inhibition can lead to increased formation of micronuclei (36).

The association of viral DNA with chromosome pulverization and chromothripsis has been previously suggested (37, 38). We screened aSCLC sequences for a wide range of human viruses by tNGS (39) and found no viral DNA in any samples.

Finally, chromothripsis has been associated with telomere dysfunction, resulting from either upregulation of telomerase reverse transcriptase (TERT; ref. 34) or through a repair-based pathway called alternative lengthening of telomeres (40). No *TERT* overexpression was identified in aSCLC cases with available RNA-seq (Supplementary Fig. S8), and no alternative lengthening of telomere footprints were identified by WGS, suggesting that these phenomena are unlikely to play a role in the development of these tumors.

### Spatial and Temporal Conservation of Chromothripsis and Associated Amplifications

**Figure 5.**
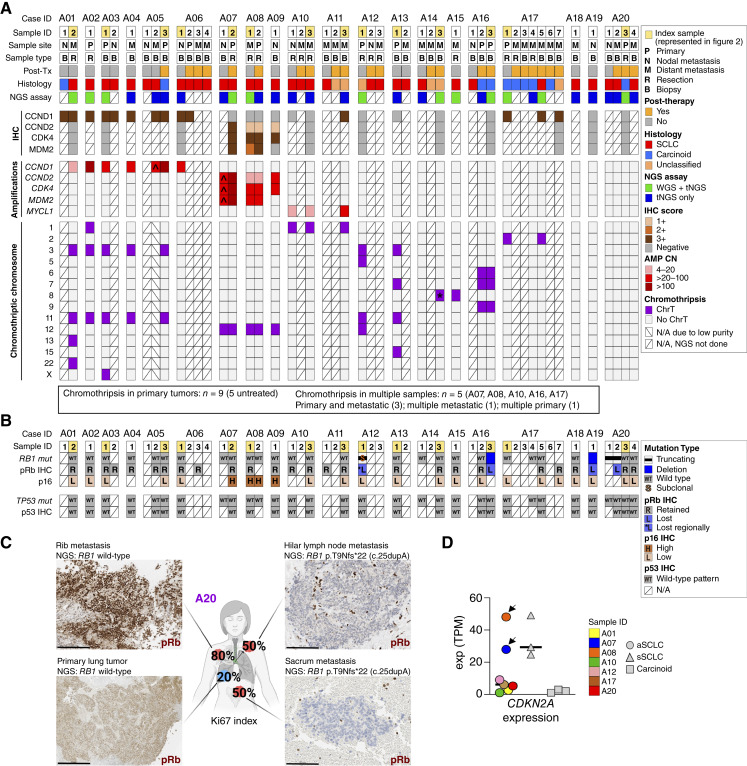
Multisample analysis from all temporally or spatially distinct samples obtained for each patient. **A,** Conservation of chromothripsis and corresponding amplifications across all samples with available data from individual patients. ^ indicates lower purity samples compared with the other sample from same patient (Supplementary Table S5); CN values in such samples may be falsely low. * indicates chromothripsis detected by WGS only, but not detected by tNGS. N/A, not available: sample without NGS or IHC. Sample numbering is chronologic (Supplementary Table S3). **B,** Multisample analysis showing *RB1* and *TP53* genomic alterations and expression of pRb, p53, and p16 by IHC, illustrating a subset of patients with acquired or subclonal *RB1* alterations/loss (details in Supplementary Table S1) and a subset with unusual patterns of p16 expression (see “Results”). **C,** Illustration of a case with acquired *RB1* mutations and loss of expression in metastatic samples. pRb-positive cells admixed with negative tumor cells in panels on the right are benign lymphocytes and stromal cells, serving as internal controls. Scale bars are 100 μm, except for the left bottom, which is 1 mm. **D,** RNA-seq for *CDKN2A*, encoding p16. Arrows indicate cases with chromothripsis of chromosome 12 and ecDNA amplification of *CDK4*. TPM, transcripts per million. (**C,** created in part with BioRender.com.)

In prior studies, chromothripsis has been postulated to represent an early causative event in tumorigenesis (17, 34), but it can also occur as an acquired event later in disease progression or in association with systemic therapy resistance (31, 34). In the aSCLC cohort, serial pathologic samples from different locations and time-points were available for 14 patients; of those, eight patients had multiple samples analyzed by tNGS (2–4 per patient), and one patient (A17) had WGS performed on both primary and metastatic samples (Figs. 1B, C, and 5A). First, all samples from individual patients were clonally related based on shared genomic alterations (Supplementary Table S5). Furthermore, they exhibited concordant presence or absence of chromothripsis involving identical chromosomes, as well as concordance in associated gene amplifications and overexpression by IHC (Fig. 5A). Lastly, the presence of chromothripsis was unrelated to the administration of systemic therapy. This indicates that in our cohort, chromothripsis represents an early and stable event that is conserved temporally and spatially through disease progression.

To further interrogate the timing of chromothripsis and associated amplifications in the clonal evolution of aSCLC, we assessed the ratio of duplicated to nonduplicated mutations within amplified regions using MutationTimeR (41). Across all samples, all or nearly all chromothripsis-associated amplifications were estimated to occur early in tumorigenesis (Supplementary Table S11), in line with our multisample data.

We next reviewed the matched NGS data for two patients with chromothripsis and histotype heterogeneity, for whom sequencing was performed on primary lung tumor samples with carcinoid histotype and metastatic samples with SCLC histotype (A05 and A17). This demonstrated matching chromothripsis patterns in primary and metastatic tumors (Supplementary Fig. S11A and S11B), and no acquired unique mutations in established oncogenes or tumor suppressor genes in samples with the SCLC histotype (Supplementary Fig. S11C). This suggests that other events superimposed on chromothripsis, possibly epigenetic, may facilitate the carcinoid-to-SCLC phenotypic transition, which, interestingly, parallels the findings in adenocarcinoma-to-SCLC plasticity where transition to SCLC phenotype is thought to be mediated by epigenetic reprogramming rather than additional genomic events (9, 10, 42).

### Multimodal Cell-Cycle Deregulation in aSCLC

Given that chromothripsis-associated amplifications were centered on upstream regulators of pRb protein (CDK4, D-type cyclins), we further explored cell-cycle deregulation using integrated DNA, RNA, and IHC results. As summarized in Supplementary Fig. S12A and S12B, pRb pathway deregulation at the DNA, mRNA, and/or protein levels was evident in 75% of the samples in this cohort.

The pRb pathway in aSCLC was further investigated via gene pathway analysis by RNA-seq, which revealed major upregulation of the proproliferative G2M checkpoint and E2F target signatures in aSCLC, at a level markedly exceeding that found in a control set of carcinoid tumors and in some cases reaching the level seen in conventional sSCLC (Supplementary Fig. S12C).

Interestingly, in the multisample analysis, we identified acquired *RB1* alterations in two patients with aSCLC (A16, A20; Fig. 5B); in both cases, *RB1* was wild-type and expressed in primary lung tumors, whereas metastatic/subsequent sample(s) exhibited acquired, private *RB1* mutations and concurrent loss of pRb expression (Fig. 5C). In addition, one primary tumor (A12) exhibited a subclonal *RB1* mutation [cancer cell fraction (CCF) 71%] with a corresponding subclonal loss of pRb expression by IHC in ∼70% of the tumor cross-sectional area. In contrast, in conventional SCLC, *RB1* mutations/loss are consistently clonal events (4). The functional significance of acquired and subclonal *RB1* alterations in aSCLC is unclear, given that there was no overt escalation in the proliferation rate associated with pRb loss (Fig. 5C). It is possible that *RB1* inactivation in these samples plays a noncanonical role outside of E2F-mediated cell-cycle control, as has been suggested for other cancer types that exhibit acquired *RB1* alterations later in disease progression (43).

In prior studies, expression of pRB and p16 was found to be consistently reciprocal in conventional SCLC (12). Interestingly, some aSCLC exhibited a unique disjoining of this reciprocity by IHC (Fig. 5B) and RNA-seq (Fig. 5D). First, all cases with chromothripsis on chromosome 12 were paradoxically p16^high^ despite expressing a wild-type pRB (A07, A08, and A09; Supplementary Fig. S2 illustrates case A08). We hypothesize that in such cases, posttranslational inhibition of pRB by ecDNA-amplified *CDK4* may be so extreme that it results in a pRb null-like state, leading to reciprocal p16 overexpression. Intriguingly, p16 overexpression was similarly noted in liposarcomas lacking *RB1* alterations but harboring ecDNA-based *CDK4* amplification (44). Also, aSCLC samples with acquired or subclonal *RB1* mutations, remarkably, lacked p16 overexpression. This could reflect epigenetic silencing of *CDKN2A*–the gene encoding p16–in the distinct precursors of aSCLC, in line with the reported *CDKN2A* promoter methylation in carcinoids/NETs (45), which, as we discuss next, may represent putative progenitors of aSCLC. Overall, these findings further highlight unique features of pRb pathway deregulation in aSCLC compared with conventional SCLC.

Lastly, unlike *RB1*, *TP53* remained consistently wild-type in multisample analysis. Notably, in cases with chromothripsis on chromosome 12, co-amplification of *MDM2* (a potent p53 antagonist), together with *CDK4* and *CCND2* (pRb antagonists), may represent a full phenocopy of the *TP5*3 and *RB1* genomic co-inactivation in conventional SCLC.

### Histogenetic Relationships in aSCLC versus SCLC in Never-Smokers with *RB1*^–^/*TP53*^–^

**Figure 6.**
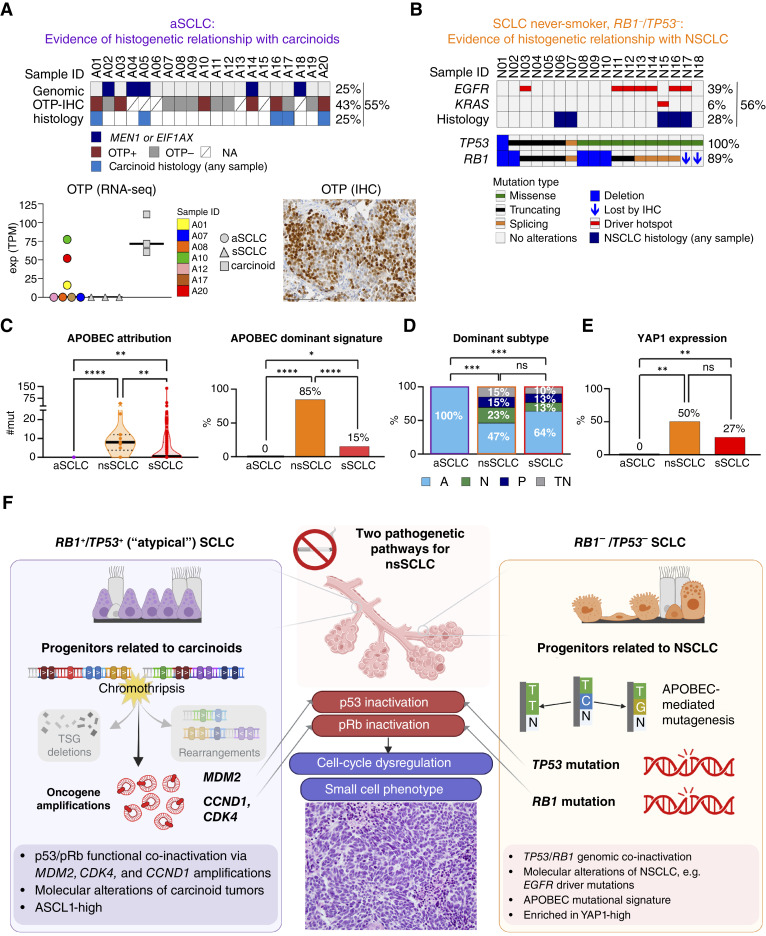
Comparison of aSCLC vs. *RB1*^−^/*TP53*^−^ nsSCLC: the dual model of SCLC pathogenesis in never-smokers. **A,** Lines of evidence for a histogenetic relationship between aSCLC and carcinoids or their progenitors, including histologic evidence of carcinoid histotype in at least one sample (see also Figs. 1C and 5A), expression of a lung carcinoid–specific gene OTP by IHC, or the presence of genomic alteration characteristic of carcinoids. Bottom, expression of OTP by RNA-seq and IHC. N/A, OTP IHC not available. Scale bar in OTP image: 100 μm. **B,** Lines of evidence for a histogenetic relationship of nsSCLC with NSCLC. **C,** Distribution of APOBEC mutational signature in aSCLC, nsSCLC, and sSCLC. See also Supplementary Fig. S5 and “Methods” for details. **D,** Expression of transcriptional subtype markers and (**E**) YAP1 by IHC. A, ASCL1-dominant; N, NEUROD1-dominant; P, POU2F3; TN, triple-negative. Subtype marker data were available for 15 aSCLC, 13 nsSCLC, and 142 sSCLC. **F,** Conceptual diagram depicting dual pathogenetic pathways underlying nsSCLC , highlighting carcinoid–SCLC pathway associated with chromothripsis vs. NSCLC–SCLC pathway associated with *EGFR* mutations and APOBEC signature. TSG, tumor suppressor genes. (**F,** created in part with BioRender.com.)

The observation that five patients with aSCLC had both SCLC and low-proliferative carcinoid histotypes detected suggested the notion that these SCLC have a histogenetic relationship with lower-grade NETs. We also found that additional aSCLC, including those without evidence of carcinoid histology, expressed orthopedia homeobox protein (OTP) by IHC and RNA-seq (Fig. 6A)—a marker that is uniquely expressed in a subset of pulmonary carcinoids, but not in conventional SCLC (46). Overall, based on histopathology, OTP expression, and/or presence of genomic alterations that, although not entirely specific, are highly characteristic of carcinoid tumors (*MEN1* and *EIF1AX*), 55% of aSCLC exhibited features of a histogenetic relationship with pulmonary carcinoids.

Among those five patients with SCLC and carcinoid histotypes, two patients had completely resected primary lung tumors (A17 and A20), and both were entirely composed of lower-proliferative carcinoid histology (Supplementary Figs. S3 and S4), suggesting that at least in some cases, aSCLC may arise through major clonal selection or dedifferentiation during metastatic progression. Also notable is that some patients with aSCLC had only SCLC histotype identified in all samples. Although this could be a result of limited sampling by small biopsies in some cases, three patients (A02, A07, and A08) had completely resected primary tumors composed entirely of SCLC histology. This conversely suggests that some aSCLC may arise via divergence at an early progenitor stage, similar to the phenomenon recognized among molecularly defined dedifferentiated sarcomas that may lack detectable differentiated components (47). Overall, we document a histogenetic relationship with carcinoids in the majority of aSCLC, with a spectrum of pathologic manifestations, that may reflect plasticity occurring at different time-points of disease evolution.

We next aimed to better characterize the differences in the genomic and clinicopathologic characteristics of aSCLC versus the *de novo* SCLC in never-smokers harboring *RB1* and *TP53* genomic mutations (nsSCLC-*RB1*^−^/*TP53*^−^; *n* = 18). In contrast to aSCLC, none of those tumors exhibited features of chromothripsis based on the lack of oscillating CNAs by tNGS. Instead, 56% of nsSCLC-*RB1*^−^/*TP53*^−^ harbored either canonical *EGFR* (39%) or *KRAS* (6%) mutations and/or displayed histologic components of NSCLC, most commonly in the form of adenocarcinoma (Fig. 6B). Furthermore, nsSCLC-*RB1*^−^/*TP53*^−^ was enriched in the apolipoprotein B mRNA-editing enzyme catalytic polypeptide-like (APOBEC) mutagenesis signature, in contrast to the lack of this signature in aSCLC (Fig. 6C; Supplementary Figs. S5B and S5C). These data support and expand on prior observations on the association with *EGFR* mutations, adenocarcinoma histologic components, and APOBEC mutagenesis in *RB1*^−^/*TP53*^−^ nsSCLC (10, 42, 48, 49) and highlight the contrast between these cases and aSCLC.

Last, we compared aSCLC and nsSCLC-*RB1*^−^/*TP53*^−^ for the distribution of ASCL1, NEUROD1, and POU2F3—recently identified markers of transcriptional subtypes in SCLC (Fig. 6D; ref. 50). Strikingly, all evaluated aSCLC were exclusively ASCL1-positive, suggesting a specific relationship between aSCLC and an ASCL1-expressing subset of carcinoids (51). In contrast, nsSCLC-*RB1*^−^/*TP53*^−^ had comparable distribution of transcriptional subtypes to that of sSCLC. YAP1—a marker associated with NSCLC-to-SCLC plasticity (52)—was enriched in nsSCLC-*RB1*^−^/*TP53*^−^, whereas it was entirely absent in aSCLC (Fig. 6E). These data further support the distinct properties of aSCLC compared with those of other SCLC.

In aggregate, our data suggest a model of SCLC pathogenesis in never-smokers involving two distinct plasticity-mediated pathways (Fig. 6F)—one, known from prior studies but expanded in our cohort, with a histogenetic link to NSCLC precursors, where conversion to SCLC occurs in *RB1*^−^/*TP53*^−^ background in association with APOBEC mutagenesis, and the other, a novel pathway, with a histogenetic link to carcinoid precursors, where the plasticity occurs in an *RB1*^+^/*TP53*^+^ background through chromothripsis-mediated amplification of oncogenes, some of which have key roles in pRb and p53 suppression.

### Survival, Treatment Outcomes, and Expression of Therapeutic Biomarkers in aSCLC

**Figure 7.**
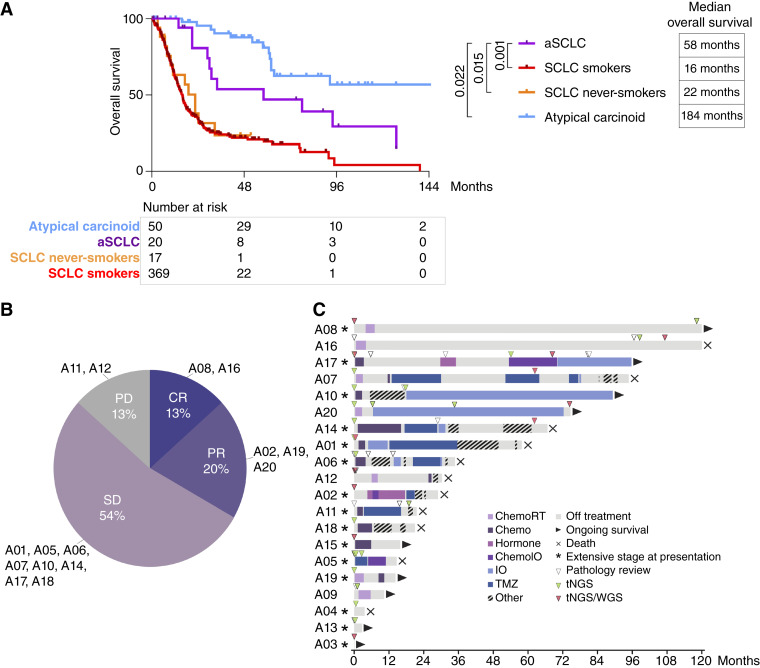
Outcome and treatment responses. **A,** Kaplan–Meier analysis of the disease-specific overall survival assessed from the time of diagnosis. **B,** Pie chart summarizing radiologic treatment response to platinum-based chemotherapy received in any line of therapy. **C,** Swimmer plot summarizing treatment modalities used and time-on-treatment, with time of pathologic samples collected and analysis performed indicated. CR, complete response; PD, progressive disease; PR, partial response; SD, stable disease.

Having identified the highly distinct genomic and clinicopathologic features of aSCLC compared with other SCLC, we next sought to characterize the clinical outcomes and treatment responses associated with this newly defined entity. The survival of patients with aSCLC was compared with that of patients with other types of SCLC (sSCLC and nsSCLC-*RB1*^−^/*TP53*^−^) and atypical carcinoids—the subset of carcinoids defined by increased mitotic rate (see “Methods”); patients with typical carcinoids were excluded from this analysis because they are well established to have only rare tumor-associated mortality (53). Kaplan–Meier analysis revealed that aSCLC was associated with distinct survival characteristics, which were intermediate between those of SCLC in smokers and atypical carcinoids (Fig. 7A). In contrast, nsSCLC-*RB1*^−^/*TP53*^−^ had an outcome similar to that of smoking-related SCLC.

We also investigated the profile of therapeutic sensitivity in aSCLC, focusing on platinum-based therapies—the mainstay first-line therapy for SCLC. Of the 15 patients with available response data for platinum-based systemic therapy, complete or partial responses were achieved in 13% and 20% of patients, respectively (Fig. 7B), which is well below the historical rates expected for conventional SCLC (70%; ref. 1). Using time-to-next treatment as a surrogate of clinical benefit, a subset of patients seemed to exhibit sensitivity to immune checkpoint inhibitors, as reflected by the length of treatment of 2 to >5 years for three of the five treated patients (Fig. 7C). An additional four of the six patients treated with TMZ had >10 months on treatment, ranging up to 2 years. The long time on treatment for these two therapies stands in contrast to the clinical experience with standard patients with SCLC and suggests that distinct pathways of treatment sensitivity may be present in aSCLC. The cohort size was too small to evaluate specific genomic or clinicopathologic features associated with survival and treatment outcomes, although we note that patients with longest time on TMZ (A01 and A07) had the lowest expression of O^6^-methylguanine-DNA methyltransferase by RNA-seq—the marker that is inversely associated with TMZ sensitivity (Supplementary Fig. S13A; ref. 54).

Several targeted therapies are currently under clinical investigation for SCLC, including agents directed at DLL3 (55) and SEZ6 (56). To determine whether patients with aSCLC might be candidates for these therapies, we analyzed the expression of these markers in samples with available tissue. All evaluated samples (*n* = 9) exhibited high levels of DLL3 (H score 200–300; mean 278) and SEZ6 (H score 140–300; mean 240; Supplementary Table S2; Supplementary Fig. S13B). The high level of DLL3 expression is in line with consistently high ASCL1 expression in aSCLC, as the *DLL3* gene is a downstream target of ASCL1 (57).

## Discussion

Here, we provide the first detailed genomic and clinicopathologic description of a rare subtype of SCLC that lacks concomitant *RB1* and *TP53* inactivation and arises in the absence of smoking-induced carcinogenesis. We identify extensive chromothripsis with recurrent ecDNA amplification of several oncogenes involved in the regulation of pRb and p53 as a hallmark feature in these cases and MSI as an alternative genomic context. Furthermore, we identify a histogenetic link between these tumors and pulmonary carcinoids, suggesting a new pathway for the development of SCLC via progression from lower-grade NETs or their progenitors. This study defines aSCLC as a novel entity among lung cancers, highlighting its exceptional etiology, distinct clinicopathologic properties, and unique therapeutic vulnerabilities.

Since its initial description in 2011, chromothripsis has emerged as a major driver of tumorigenesis that can exert a profound impact on tumor genomes by generating diverse genomic alterations (17). A common consequence of chromothripsis is oncogene amplification, which often occurs as ecDNA—the autonomously replicating unit that enables the accumulation of a remarkably high CN of amplified genes (31, 58). Similar to aSCLC, *CCND1*, *CDK4*, and *MDM2* are among the most common amplifications associated with chromothripsis across tumor type (18). In addition to ecDNA, we also found evidence of *CCND1* upregulation through putative enhancer hijacking resulting from chromothripsis-mediated rearrangement of regulatory elements, highlighting the diversity of mechanisms by which chromothripsis may contribute to cancer development. In a minority of cases, established driver gene(s) targeted by chromothripsis could not be identified; it is possible that cumulative effect of alterations in multiple genes could underly carcinogenesis in such tumors.

Chromothripsis has been identified across a wide spectrum of cancer types, with a particularly high prevalence in sarcomas and gliomas (18, 34). Although identified as a hallmark feature of aSCLC, chromothripsis is not unique to this subset of SCLC. In fact, the initial description of chromothripsis included an *RB1*^*−*^/*TP5*3^*−*^ SCLC cell line (SCLC-21H), which harbored chromothripsis involving chromosome 8 with ecDNA amplification of *MYC* (17, 59). Furthermore, recent studies have identified ecDNA amplification of *MYC* paralogs in a minority of *de novo* SCLC tumor samples (60) and posttreatment SCLC models (61), although the prevalence of underlying chromothripsis in such cases remains to be determined. In our analysis of a set of published whole genomes of *RB1*^*–*^/*TP5*3^*–*^ SCLC, chromothripsis was indeed identified in a minority of cases but was substantially more limited in extent than in aSCLC. Overall, although chromothripsis and ecDNA oncogene amplification are not exclusive to aSCLC, this cohort is distinguished by its high prevalence and extent, recurrent involvement of chromosomes 3, 11, and 12, and *RB1*^*+*^/*TP5*3^+^ context with low number of other mutations, suggesting that in these tumors, chromothripsis represents a central driver of tumorigenesis.

Analysis of chromothripsis and its clinicopathologic significance at scale has been hampered by the lack of established methods for the detection of this phenomenon in panel NGS assays utilized for clinical sequencing. To date, large-scale studies on chromothripsis have been based primarily on WGS, utilizing integrated assessment of SVs and CNAs (18, 34); although CNA only–based approaches have also been applied to whole-genome and whole-exome sequencing (62, 63) or genomic array platforms (61). To the best of our knowledge, this study is the first to demonstrate the feasibility of detecting chromothripsis in a panel NGS assay based on a distinctive oscillating CNA pattern. Although a larger set of samples is needed to fully validate the performance metrics, this advance should facilitate wider recognition of chromothripsis in NGS panel sequencing.

Currently, the understanding of lung neuroendocrine cancer centers on SCLC and carcinoids representing entirely unrelated tumor entities, occurring in distinct patient populations (younger, never-smokers for carcinoids vs. older smokers for SCLC), and characterized by highly distinct genomic profiles, most notably separated by *RB1* and *TP53* genomic status and TMB (12). Here, we identified that most of *RB1*^+^/*TP53*^+^ tumors that have histomorphology of *bona fide* SCLC exhibited evidence of a histogenetic link with carcinoids, including harboring genomic alterations and marker expression characteristic of pulmonary carcinoids, and exhibiting co-occurring carcinoid histotype in some cases. Small cell transformation—that may occur in *de novo* tumors or as a form of acquired resistance to therapy—is a well-established phenomenon in several organs, including lung and prostate adenocarcinomas (64), in line with the concept of small cell carcinoma representing a convergent phenotype that may arise from a variety of precursors (65). Here, we add carcinoid–SCLC transition as a rare form of plasticity associated with small cell phenotype.

Pulmonary carcinoids are generally indolent tumors, but a subset can metastasize and behave aggressively (12). Aggressive subsets of carcinoids have been suggested based on gene expression characteristics (51), genomic features (66), and high TERT expression (67). We postulate that chromothripsis may represent a novel tractable risk factor for carcinoid progression and dedifferentiation. Previously, isolated instances of chromothripsis in lung carcinoids were reported, including cases with chromothripsis on chromosomes 3, 12 and 13 (68), 2, 11 and 20 (68), 11 and 20 (51) and 11 (69). Only limited clinicopathologic information is available for most of these cases, but some exhibited aggressive clinical behavior (68).

In our series, all patients with *RB1*^*+*^/*TP53*^*+*^ SCLC were never or light smokers. However, in the study of George and colleagues (2), one of the two patients with chromothripsis-associated *RB1*^*+*^/*TP53*^*+*^ SCLC was reported to have a 30 pack-year smoking history. Thus, in aggregate, although apparently uncommon, tobacco exposure may not preclude chromothripsis-mediated pathogenesis in SCLC.

In this study, we contrasted aSCLC with *de novo* SCLC in never smokers harboring *RB1* and *TP53* genomic alterations—another rare and incompletely characterized subset of SCLC. The genomic and pathologic data generated here support predominant adenocarcinoma-to-SCLC plasticity in this subset and its association with *EGFR* mutations and APOBEC mutagenesis and contrast this pathway with carcinoid-to-SCLC plasticity associated with chromothripsis in aSCLC. A translational implication of this model is that *RB1*/*TP53* mutation status—which can be assessed by routine immunohistochemical methods—may serve as surrogate markers for putative tumor progenitors in the population of never smokers with SCLC. This is clinically relevant because aSCLC is associated with a distinct prognosis and might ultimately define a category with distinct therapeutic approaches.

Our study suggests several potential therapeutic vulnerabilities in aSCLC based on the unique genomic underpinnings of these tumors. These may include agents under clinical development that target ecDNA-based oncogene amplification (70), as well as agents targeting amplification and overexpression of CDK4 (71) and MDM2 (72). Furthermore, consistently high expression of DLL3 and SEZ6 suggests that these patients could benefit from the emerging therapies targeting these cell surface determinants (55, 56). Such targets may be of particular importance given the relative platinum insensitivity of aSCLC. The apparent efficacy of TMZ is also notable, as this agent is active in both SCLC (54) and lung carcinoids (73), given the dual histologic characteristics in this cohort.

In conclusion, here we describe a new pathway for the development of SCLC mediated predominantly by chromothripsis in tumors with a histogenetic link with lower-grade carcinoid tumors or their progenitors. This study establishes a novel concept in lung tumorigenesis with potential therapeutic implications.

## Methods

### Study Design

This study was approved by the Memorial Sloan Kettering Cancer Center (MSKCC) Institutional Review Board (IRB). In accordance with the Declaration of Helsinki, all patients included in this study signed a written informed consent form following the IRB’s recommendations. All patients included in the study had tumors analyzed prospectively by MSK-IMPACT as part of routine clinical care at the MSKCC. A detailed review of the demographic, radiologic, pathologic, and clinical information was performed retrospectively. Pathology slides were retrieved and re-reviewed. If sufficient residual DNA or formalin-fixed paraffin-embedded (FFPE) tissue was available, the samples were further analyzed by WGS, RNA-seq, FISH, and additional IHC. For patients with multiple samples, WGS was performed on the chronologically earliest sample or based on sufficiency for additional testing. For comparison with the study group, cohorts of SCLC (4) and pulmonary carcinoids that were prospectively sequenced using MSK-IMPACT were included in the analysis.

### Clinicopathologic Assessment

Clinical patient characteristics were annotated by reviewing the electronic medical record. The baseline characteristics included age, sex, tobacco smoking history, date of diagnosis, pathology sample site, and pathology sample type. Radiology data were reviewed to collect information on the metastatic site distribution at presentation. Smoking history was collected from the patient-completed smoking questionnaire. Pack-years of smoking were derived as follows: [(average number of cigarettes smoked per day/20) X years of smoking]. Never-smokers were defined as patients who had smoked <100 cigarettes, and light smokers were defined as those who had a ≤10 pack-year smoking history.

Tumor classification was performed according to the criteria in the 2021 World Health Organization classification of thoracic tumors (74). All tumors underwent central pathologic review by pathologists with expertise in thoracic tumors (NR, CFA, and JC). The criteria for SCLC included undifferentiated morphology with high a nuclear/cytoplasmic ratio and nearly imperceptible cytoplasm, cell molding, mitotic count of >10 per 2 mm^2^ (if sufficient well-preserved tissue available to perform the counts) and Ki67 proliferation index of ≥50%, commonly associated with extensive necrosis. The criteria for carcinoids included a well-differentiated morphology composed of uniform cells with lower nuclear/cytoplasmic ratios and readily visible cytoplasm, mitotic count of ≤10 per 2 mm^2^, and absence of extensive necrosis. For survival analysis, carcinoids were classified as typical or atypical using the World Health Organization criteria: <2 mitoses per 2 mm^2^ and no necrosis for typical carcinoids, and ≥2 mitoses per 2 mm^2^ and/or focal necrosis for atypical carcinoids. Samples that were too crushed or poorly preserved to evaluate the morphology, or that had equivocal morphologic features, were regarded as unclassified. For all patients, pathologic specimens and clinical records were reviewed in detail to exclude the possibility of an alternative tumor type or nonpulmonary origin.

### IHC

IHC was performed by previously established and validated protocols, as summarized in detail in Supplementary Table S12. Primary antibodies included synaptophysin (SNP88, BioGenex), chromogranin A (LK2H10, Ventana), CD56/NCAM/neural cell adhesion molecule (MRQ42, Cell Marque), INSM/-nsulinoma-associated protein 1 (A-8, Santa Cruz Biotechnology), Ki67 (MIB1, Dako), OTP/orthopedia homeobox protein (EPR22178-17, Abcam), ASCL1 (24B72D11.1, BD Biosciences), NEUROD1 (EPR17084, Abcam), POU2F3 (6D1, Santa Cruz Biotechnology), YAP1 (63.7, Santa Cruz Biotechnology), DLL3 (SP347, Ventana), SEZ6 (SC17.14, Creative Biolabs), cyclin D1 (SP4, Lab Vision), cyclin D2 (M20, Santa Cruz Biotechnology), Mdm2 (IF2, Millipore), Cdk4 (CDS-156, BD Biosciences), pRb (13A10, Leica), p53 (D07, Ventana), and p16 (E6H4, Ventana). Transcriptional subtype (SCLC-A, -N, -P, -TN/triple negative) was assigned based on the predominant expression of ASCL1, NEUROD1, POU2F3, or triple-negative by IHC, respectively, as described previously (52). For semi-quantitative scoring of DLL3 and SEZ6, the histologic (H) score was derived by multiplying the intensity of staining (1+ weak, 2+ moderate, and 3+ strong) by the percentage of cell staining (1%–100%), yielding H scores from 0 to 300. The Ki67 proliferation index was assessed as the percentage of positive cells in hot-spot areas—regions with the highest Ki67 rate counted in at least 500 tumor cells (75).

### Survival and Treatment Outcome Analysis

Data on patient treatments and outcomes were collected by reviewing electronic medical records, including treatments administered and best response to platinum/etoposide, date of death, or last follow-up. Disease-specific overall survival was estimated from the date of diagnosis to the date of documented death from disease or the last follow-up using the Kaplan–Meier approach. For comparative survival analysis, control groups of SCLC in smokers and atypical carcinoids were generated, consisting of consecutively encountered patients with available clinical follow-up. For the swimmer plots, treatment times were depicted from the first administration date of one therapy to a next therapy, counting maintenance therapies (such as platinum/etoposide/atezolizumab followed by maintenance atezolizumab) as one regimen. Imaging studies and reports were manually reviewed to generate a real-world response rate by comparing on-treatment scans to pretreatment scans. Patients were considered to have partial response, complete response, stable disease, or progressive disease on the basis of clinician interpretation of the change in disease burden on subsequent scans from the first pretreatment scans.

### Targeted NGS by MSK-IMPACT

Genomic sequencing was performed on tumor DNA extracted from FFPE tissue, and normal DNA was sequenced in all patients using the FDA-authorized MSK-IMPACT–targeted sequencing panel using methods and analysis as previously described (5, 6). Briefly, the MSK-IMPACT assay is a clinically validated FDA-authorized custom hybridization capture-based platform that sequences the entire coding region and select noncoding regions of 341 (v3–1 sample), 468 (v5–2 samples), or 505 (v6–17 samples) genes for the detection of SNVs, indels, CNAs, and select SVs (gene list provided in Supplementary Table S13). Somatic alterations were classified as oncogenic, likely oncogenic, or unknown using OncoKB (19, 76).

TMB was calculated as the number of nonsynonymous mutations in canonical exons per megabase. Tumor purity was estimated by FACETS and the “hisense” solution is reported, cval parameter = 50. MSI was analyzed using MSIsensor (https://github.com/ding-lab/msisensor) from the MSK-IMPACT sequencing data. A minimum of 800 loci and tumor-normal matched sequencing were required for MSI evaluation. Tumors with MSIsensor scores of <3, ≥3 to <10, and ≥10 were classified as MS stable, MSI indeterminate, and MSI-H, respectively, using previously validated cutoffs (77). MSI indeterminate tumors were adjudicated by MiMSI, an independent algorithm for MSI calling based on multiple instance learning (https://www.biorxiv.org/content/10.1101/2020.09.16.299925v1.full.pdf).

To assess subclonal mutations, CCF was estimated for selected SNVs as a function of variant allele frequency (VAF), tumor purity (p), and allelic CN state, as previously reported: VAF(CCF) = p × CCF/[CN_diploid × (1 − p) + CN_mut × p] and using a binomial distribution and maximum likelihood estimation normalized to produce posterior probabilities (78). CN_mut was calculated using the expected number of copies for each mutation generated based on the observed VAF and local CN (via FACETS, see below; ref. 79); a CCF of ≥0.8 was regarded as clonal or near-clonal.

### Assessment of *RB1* and *TP53* by Integrated Genomic and IHC Analysis

The screening of consecutive SCLC analyzed by MSK-IMPACT for *RB1* and *TP53* status was performed using an integrated approach utilizing genomic alterations and expression of pRb and p53 proteins by IHC, respectively. Based on prior work (4), for cases lacking detectable *RB1* (NM_000321) genomic alterations by MSK-IMPACT routine clinical pipeline (which covers all exons of *RB1* gene in all versions of MSK-IMPACT and 5′ untranslated region and introns 6, 8, and 23 in latest version V6), manual review was performed to identify noncanonical splice-site mutations. Also, pRb IHC was performed, and cases with complete loss of pRb protein expression were classified as *RB1*-deficient (*RB1*^−^), whereas only those lacking *RB1* genomic alterations and exhibiting retained pRb expression were designated as *RB1*-proficient (*RB1*^+^). Also, as reported previously (4), we assessed the expression of D-type cyclins and p16^INK4A^—the upstream pRb regulators—to further corroborate pRb proficiency, which was supported by D-type cyclin^high^ (H score >50) and/or p16^low^ (H score <100) profile.

p53 IHC was also performed for all cases lacking *TP53* (NM_000546) genomic alterations by MSK-IMPACT (which covers all exons of *TP53* in all versions of MSK-IMPACT and 5' untranslated region in latest version V6) to confirm the wild-type expression pattern. Using the standard criteria, p53 mutant/aberrant pattern included any one of the three patterns: strong nuclear intensity in 80% to 100% tumor cells (overexpression pattern, reflecting aberrant degradation of p53, usually resulting from missense *TP53* mutations), complete lack of immunoreactivity (null pattern, reflecting degradation of p53 harboring truncating *TP53* mutations), or strong cytoplasmic reactivity only (reflecting mutations disrupting nuclear localization domain), whereas p53 wild-type pattern was defined as any staining other than the three mutant patterns (80, 81).

Cases lacking *RB1* or *TP53* genomic alteration but with insufficient tissue for IHC confirmation were excluded.

### Analysis of CNAs in MSK-IMPACT

The CNAs were evaluated by MSK-IMPACT using the coverage-based method (82). The FACETS algorithm (83) was also applied to define CN states, the total CN in gene amplifications, chromosome-level alterations, and screening for chromothripsis. FACETS was run on matched normal mode using two critical segmentation values (CVAL): a “purity” output using a CVAL = 150 and “hisens” output with CVAL = 50. Other refitting parameters such as diplogR and minNhet were adjusted on a case-by-case basis (83).

To assess potential chromothripsis by MSK-IMPACT, FACETS hisens output plots were manually inspected to identify chromosomal segments oscillating between predominantly two CN states. A minimum number of five consecutive oscillating uniformly sized segments with estimated CCF ≥50% and matched patterns between plots (log-ratio CN, OR CN, and integer CN) were considered suggestive of chromothripsis. A threshold of five consecutive oscillations was chosen to minimize the risk of overcalling chromothripsis in cases in which two gene-level amplifications or deletions occurred in close proximity. Chromothripsis calls and method performance were evaluated by comparison with CN segments and SVs using WGS in cases with available material (see “Results”).

### Analysis of Mutational Signatures in MSK-IMPACT

The mutational spectra of the SNVs were calculated using a custom algorithm designed in-house (https://github.com/mskcc/DeepSig/). To obtain maximal sensitivity for single base substitution (SBS) signatures in SCLC which has no established signature catalog, we first employed *de novo* signature detection using a combined panel of 11 WGS samples from this study, 10 WGS samples that were *RB1*/*TP53* deficient from George and colleagues (2; processed through an in-house pipeline TEMPO, https://github.com/mskcc/tempo), and SNV calls from 101 additional SCLC samples available from the supplementary table in George and colleagues (2). Briefly, using the Bayesian marginal likelihood method, we determined the most likely number of signatures present, 10. Next, signature decomposition was performed on the optimal number of signatures, and *de novo* signatures were subsequently annotated to known reference signatures using cosine similarity. *De novo* signatures with cosine similarity >0.7 to known Cosmic V3 signatures were called as the Cosmic signatures. Signatures with a common etiology were merged, e.g., SBS4 and SBS92; SBS31 and subtypes. Significance thresholds (alpha) were defined for each signature as follows: SBS44: 1e–4; SBS11: 1e–3; SBS40: 1e–3; all others: 5e–2. Dominant signatures were assigned to each sample as the signature with the largest mutational attribution. Only samples with ≥5 SNVs and those that had a matched normal were included in the analysis.

### DNA Virus Read Detection in MSK-IMPACT

The presence of DNA viruses in tumor samples was determined by the analysis of off-target reads, as previously described (39). Briefly, all tNGS reads were aligned to the human genome (hg19). Paired unmapped reads from the processed BAM files were extracted into the FASTA files. Unmapped reads from each sample were queried for viral content using blastn 2.9.0+ (parameters: strand both, word_size 28, e-value 1 e–10, perc_identity 90) and mapped to the genomes of selected human DNA viruses (Epstein-Barr Virus types 1–2, Human Herpesvirus 6 types 1–8, Merkel cell polyomavirus, and human papillomavirus types 3, 5, 8, 9, 20, 21, 29, 33, 36, 45, 62, 71, 72, 74, 77, 81, 82, 86, 92, 105, 107, 115, 117, 118, 147, 150, 152, 174, and 178) from the National Center for Biotechnology Information Virus database. Each paired read that aligned with >90% identity was quantified as a read for the specific virus. Samples with >2 paired reads for a specific virus were considered positive.

### Assessment of Germline Variants

To interrogate germline variants, a modified sequencing pipeline for paired tumor/normal MSK-IMPACT was utilized, which has been validated for clinical use in the context of an IRB-approved protocol (84), which covers 90 well-established cancer predisposing genes for pathogenic and likely pathogenic germline variants (listed in Supplementary Table S13). This analysis was performed for 15 evaluable patients. Furthermore, two additional patients underwent clinical germline testing using peripheral blood, performed using a New York State Department of Health–approved germline test covering up to 90 hereditary cancer predisposition genes (82, 85).

### WGS

After PicoGreen quantification and quality control by Agilent TapeStation, 293 to 500 ng of genomic DNA was sheared using an LE220-plus Focused-ultrasonicator (Covaris, catalog # 500569), and sequencing libraries were prepared using the KAPA HyperPrep Kit (Kapa Biosystems KK8504) with modifications. Briefly, libraries were subjected to a 0.5× size selection using aMPure XP beads (Beckman Coulter, catalog # A63882) after postligation cleanup. Libraries were either not amplified by PCR and were pooled equivolume and quantitated based on their initial sequencing performance or were amplified with five cycles of PCR and pooled equimolar. Samples were run on a NovaSeq 6000 in a PE150 run using the NovaSeq 6000 SBS Kit and an S4 flow cell (Illumina). The average number of read pairs per sample was 1.4/1.1 billion for tumors and normal, respectively, corresponding to 102×/83× coverage. The coverage range for tumor and normal WGS samples was 96.4× to 192.1× and 59.2× to 123.6×, respectively.

The WGS data were processed and analyzed using the TEMPO pipeline (https://github.com/mskcc/tempo). Briefly, the FASTQ files were aligned to the b37 assembly of the human reference genome from the GATK (https://software.broadinstitute.org/gatk/) bundle using BWA mem (v0.7.17; http://bio-bwa.sourceforge.net/). The aligned reads were converted and sorted into BAM files using samtools (v1.9; http://htslib.org/) and marked for PCR duplicates using GATK MarkDuplicates (v3.8-1). CNAs and loss of heterozygosity were determined using the FACETS (https://github.com/mskcc/facets; ref. 83) and FACETS-suite (https://github.com/mskcc/facets-suite). The FACETS parameters (CVAL, diplogR) were adjusted on a case-by-case basis according to visual inspection. SVs were called using Manta (https://github.com/Illumina/manta; ref. 86), SvABA (https://github.com/walaj/svaba; ref. 87) and BRASS (https://github.com/cancerit/BRASS). Variants were normalized to a common representation and merged using a fixed window size of 200 bps using mergesvvcf (https://github.com/papaemmelab/mergeSVvcf). Genes were annotated as oncogenic or likely oncogenic according to the OncoKB Cancer Gene List (https://www.oncokb.org/cancer-genes, update 7/2023; ref. 19). The merged SV calls were annotated using iAnnotateSV (https://github.com/rhshah/iAnnotateSV). Telomere content and shortening was estimated using TelomereHunter (https://github.com/linasieverling/TelomereHunter; ref. 88).

### *RB1* and *TP53* Assessment by WGS

Noncoding variants identified in *RB1* and *TP53* by WGS were annotated using three algorithms: namely Combined Annotation Dependent Depletion (89), Functional Analysis through Hidden Markov Models with extended Features (90), and SpliceAI (91). Overall pathogenicity (likely pathogenic or benign) for a variant was determined based on the majority consensus from all three prediction algorithms.

### Assessment of Chromothripsis in WGS

Merged SV and CNA calls from the FACETS were inputted in ShatterSeek (18), and regions were determined to be chromothripsis if one or more of the following criteria were met: (i) At least six interleaved intrachromosomal SVs, seven contiguous segments oscillating between two CN states, the fragment joins test, and either the chromosomal enrichment or the exponential distribution of the breakpoints test. (ii) At least three interleaved intrachromosomal SVs and four or more interchromosomal SVs, seven contiguous segments oscillating between two CN states, and the fragment joins test. (iii) At least 40 interleaved intrachromosomal SVs and the fragment joins test. (iv) At least 100 SVs (intrachromosomal+ interchromosomal) and at least five contiguous segments oscillating between two CN states. (v) At least six interleaved intrachromosomal SVs, four, five, or six adjacent segments oscillating between two CN states, the fragment joins test, and either the chromosomal enrichment or the exponential distribution of breakpoints test. If a region only passed criterion 5, it was regarded as “low density” for comparison with tNGS chromothripsis calling. Circos plots to visualize SV and CN were created using signature.tools.lib in R (https://github.com/Nik-Zainal-Group/signature.tools.lib).

### Integrative Analysis of SV Breakpoints and Associated CNAs by WGS

To infer mechanistic patterns in the WGS, we applied the Hartwig Medical Foundation bioinformatics pipeline for our analysis (https://github.com/hartwigmedical/hmftools; ref. 92). This pipeline was chosen because, in their PURPLE algorithm (v2.54), the boundaries of CN segments were determined by jointly analyzing the regional depth of coverage (COBALT v1.11), B-allele frequency (AMBER v3.5), and, most importantly, SVs. This integration resulted in near-complete concordance between the rearrangement breakpoints and the CN boundaries, which was pivotal in analyzing the SVs at the amplification boundaries. SVs were called primarily by GRIDSS2 (v2.12.0; ref. 93; https://github.com/PapenfussLab/rids), annotated with RepeatMasker (v4.1.2-p1; http://repeatmasker.org/) and Kraken2 (v2.1.2; ref. 94; https://github.com/DerrickWood/kraken2/), filtered by GRIPSS (v1.9), and further annotated and analyzed with LINX (v1.15; ref. 95). Complex genomic rearrangements were reconstructed, as previously described (96).

### Assessment of the Timeline of Amplifications in WGS

MutationTimeR (https://github.com/gerstung-lab/MutationTimeR) was run with default settings to estimate the timing for CN alterations in chromothriptic regions. Mutation MAF files from TEMPO were first converted to VCF (Variant Call Format) format using maf2vcf (https://github.com/mskcc/vcf2maf/blob/main/maf2vcf.pl). CN alteration segments were generated from FACETS as previously described. Subclonal cluster information was estimated using CliPP (https://github.com/wwylab/CliPP), except in sample A07 in which due to the large number of TMZ-induced mutations, computational resources limited CliPP from finishing. In this case, a placeholder for subclonal mutations at 50% purity was used per the author’s suggestion. Timing of CN gains and amplifications for key genes (e.g., *CCND1* and *CDK4*) were computed on a scale from 0 (early) to 1 (late), corresponding to the proportion of mutations before the gain.

### Assessment of Chromothripsis in Other WGS Cohorts

To compare chromothripsis events in aSCLC to other major lung cancer types, 21 WGS of *RB1*/*TP53* co-mutated SCLC were downloaded from a previous study (2). BAM files were first converted to FASTQ (GATK v4.1.9.0 SamToFastq) and then processed using the same TEMPO pipeline as the aSCLC samples. Similar to aSCLC samples, ShatterSeek was used to determine chromothripsis events across all samples with the same thresholds as previously described. Included SCLC samples IDs were as follows: S00830, S00945, S02065, S02209, S02219, S02237, S02243, S02248, S02274, S02402, S00838, S01297, S01366, S01861, S01873, S02139, S02234, S02277, S02328, S02376, and S02241.

For comparison with LUAD, WGS data were obtained from a previous study (18). Although the pipeline for processing the WGS data differed from the one used for aSCLC samples, both used ShatterSeek to obtain chromothripsis metrics, and are expected to yield comparable calls for SV and CAN used in the calling of chromothripsis. The same criteria for calling chromothripsis events in the aSCLC samples were used in calling chromothripsis in LUAD. Included LUAD sample IDs were as follows: TCGA-55-6986, TCGA-50-6597, TCGA-67-3771, TCGA-64-1680, TCGA-49-6742, TCGA-55-8299, TCGA-05-4398, TCGA-55-6982, TCGA-50-5930, TCGA-97-8171, TCGA-05-4420, TCGA-78-7535, TCGA-73-4666, TCGA-78-7158, TCGA-49-4486, TCGA-05-5429, TCGA-55-7281, TCGA-05-4397, TCGA-05-4395, TCGA-91-6847, TCGA-50-6591, TCGA-05-4396, TCGA-49-4512, TCGA-05-4389, TCGA-75-6203, TCGA-44-2659, TCGA-55-6972, TCGA-38-4628, TCGA-73-4659, TCGA-75-5147, TCGA-91-6840, TCGA-67-6215, TCGA-75-7030, TCGA-50-5932, TCGA-55-6984, and TCGA-64-1678.

### RNA-seq

After RiboGreen quantification and quality control by Agilent Bioanalyzer, 1 μg of total RNA with DV200 percentages varying from 30% to 69% underwent ribosomal depletion and library preparation using the TruSeq Stranded Total RNA LT Kit (Illumina, catalog # RS-122-1202), according to instructions provided by the manufacturer with eight cycles of PCR. Samples were barcoded and run on a NovaSeq 6000 in a PE100 run, using the NovaSeq 6000 S4 Reagent Kit (200 cycles; Illumina). On average, 131 million paired reads were generated per sample, and 26% of the data were mapped to the transcriptome.

Reads were aligned and processed using the MSKCC FORTE pipeline (https://github.com/mskcc/forte). Briefly, raw FASTQ files from RNA-seq paired-end sequencing were aligned to the Ensembl GRCh37 Homo sapiens release 99 transcriptomes using Kallisto (97) and filtered to remove transcripts with low counts. Gene expression levels were then calculated as transcripts per million using Sleuth (98). Fusions were determined by Arriba (97) and Fusion Catcher (99), and calls that were only found in a single caller were culled. Fusion frame was determined by the individual callers. Single sample gene set enrichment analysis was performed using the GSVA package in R (100). Limma (101) was used to apply a linear model to the data to determine pathways significantly different between the cohorts.

### FISH

FISH was performed on 5-μm sections from FFPE tissue for all evaluated cases. Additionally, FISH was performed on cells from a patient-derived xenograft corresponding to case A07 (P-0039208), established as previously described (102) under the approval of the MSKCC Animal Care and Use Committee (IACUC Protocol 04-03-009). FISH analysis was performed using a two-color *CCND1*/*Cen11* probe and a three-color *CCND2*/*CDK4*/*MDM2* probe to confirm gene amplifications detected by NGS analysis. The two-color probe mix consisted of bacterial artificial chromosome clones spanning *CCND1* (RP11-300I6 chr11: 69,453,281–69,614,785; RP11-804L21 chr11: 69,589,482–69,628,306; labeled with Red dUTP) and the centromeric repeat plasmid for chromosome 11 served as the control (clone pLC11A; labeled with Green dUTP). The three-color probe mix consisted of bacterial artificial chromosome clones spanning *CDK4* (RP11-571M6 chr12: 57,999,870–58,211,408; RP11-970A5 chr12: 58,136,289–58,353,071; labeled with Green dUTP), *MDM2* (RP11-611O2 chr12: 69,192,689–69,343,255; RP11-630N19 chr12: 69,337,168–69,510,888; labeled with Red dUTP), and *CCND2* (purchased from Empire Genomics, sequenced per proprietary; labeled with Orange dUTP). Probe labeling, tissue processing, hybridization, posthybridization washing, and fluorescence detection were performed according to the standard laboratory procedures. Slides were scanned using a Zeiss Axioplan 2i epifluorescence microscope equipped with a MetaSystems imaging system. The Metafer and Vslide modules within the system were used to generate virtual images of hematoxylin and eosin– and DAPI (4’,6-diamidino-2-phenylindole)-stained sections. The hematoxylin and eosin sections served as guides to identify corresponding tumor regions in the DAPI-stained slides. The entire hybridized area or section was scanned under a 63× objective to assess the amplification status and representative regions imaged through the depth of the tissue. Amplification was defined as ≥6 copies of the gene and further categorized as double minute (ecDNA segments often observed as paired signals) or homogeneously staining region (intrachromosomal DNA segments observed as medium to large, clustered signal).

### Quantification and Statistical Analysis

Statistical analyses were conducted using R version 4.2.3 (R Project for Statistical Computing; http://www.r-project.org/) and GraphPad Prism V10 (GraphPad Software). Fisher’s exact test and Mann–Whitney *U* test were used to assess the categorical and continuous variables, respectively. All parametric and nonparametric tests were two-tailed, with *P* < 0.05 considered statistically significant.

### Data Availability

The cBioPortal repository was created for all aSCLC samples in this study and can be accessed at https://www.cbioportal.org/study/summary?id=asclc_msk_2024. Raw data for WGS and RNA-seq are available in dbGAP: accession # phs003676.v1.p1 (http://www.ncbi.nlm.nih.gov/projects/gap/cgi-bin/study.cgi?study_id=phs003676.v1.p1).

The code used to analyze the WGS from Tempo is available at https://github.com/mskcc/tempo. The code for the reconstruction of complex genomic events can be found at https://github.com/parklab/focal-amplification. The code for mutational signature analysis can be found at https://github.com/mskcc/DeepSig/. The pipeline for the transcriptome analysis can be found at https://github.com/mskcc/forte.

## Supplementary Material

Supplementary Tables S1-S13 Supplementary Table S1. Integrated genomic and immunohistochemical results for TP53 and RB1 in aSCLC Supplementary Table S2. Demographic, clinicopathologic and immunohistochemical characteristics: Per patient data Supplementary Table S3. Samples and studies performed: Per sample data Supplementary Table S4. Clinicopathologic comparison aSCLC vs other SCLC Supplementary Table S5. MSK-IMPACT metrics and full results for all samples Supplementary Table S6. Full list of SNV by MSK-IMPACT in aSCLC Supplementary Table S7. Full list of CNA by MSK-IMPACT in aSCLC Supplementary Table S8. RB1 and TP53 non-coding variant annotation by WGS Supplementary Table S9. Full list of SV by WGS in aSCLC Supplementary Table S10. Full list of fusions by RNAseq in aSCLC Supplementary Table S11. Timing of amplifications by WGS: mutTimerR Supplementary Table S12. Immunohistochemical antibodies, protocols and scoring criteria Supplementary Table S13. List of covered genes by targeted NGS (MSK-IMPACT assay): somatic and germline.

Supplementary Figures S1-S13 Supplementary Figure S1. Detailed morphologic and immunohistochemical findings: Case A01. Supplementary Figure S2. Detailed morphologic and immunohistochemical findings: Case A08. Supplementary Figure S3. Detailed morphologic and immunohistochemical findings: Case A17. Supplementary Figure S4. Detailed morphologic and immunohistochemical findings: Case A20. Supplementary Figure S5. Mutational signatures in atypical SCLC (aSCLC), never-smoker SCLC with RB1–/TP53– (nsSCLC) and smoking-associated SCLC (sSCLC) analyzed by MSK-IMPACT. Supplementary Figure S6. Circos plots showing structural variants and copy number alterations across the genome from all cases with WGS. Supplementary Figure S7. Chromothripsis assessment by targeted NGS (MSK-IMPACT) versus WGS. Supplementary Figure S8. RNAseq for non-recurrently amplified (KRAS, ERBB3, KDM5A) or deleted (TGFBR2, ARID1A) genes on chromothriptic chromosomes and TERT. Supplementary Figure S9. Comparison of chromothripsis characteristics in aSCLC vs other major lung cancer types. Supplementary Figure S10. Chromothripsis architecture in all cases profiled by whole-genome sequencing. Supplementary Figure S11. Chromothripsis in multi-sample analysis and genomic alterations in samples with histotype heterogeneity. Supplementary Figure S12. Cell cycle and p53 pathway deregulation in aSCLC. Supplementary Figure S13. Expression of potential therapeutic markers in aSCLC.
