# Supplementary material for: Chromothripsis-Mediated Small Cell Lung Carcinoma
Source: Cancer Discov. 2024 Aug 26;15(1):83–104. doi: 10.1158/2159-8290.CD-24-0286 (PMC11726019; doi:10.1158/2159-8290.CD-24-0286)
Supplement: Supplementary Figures S1-S13 — Supplementary Figure S1. Detailed morphologic and immunohistochemical findings: Case A01. Supplementary Figure S2. Detailed morphologic and immunohistochemical findings: Case A08. Supplementary Figure S3. Detailed morphologic and immunohistochemical findings: Case A17. Supplementary Figure S4. Detailed morphologic and immunohistochemical findings: Case A20. Supplementary Figure S5. Mutational signatures in atypical SCLC (aSCLC), never-smoker SCLC with RB1–/TP53– (nsSCLC) and smoking-associated SCLC (sSCLC) analyzed by MSK-IMPACT. Supplementary Figure S6. Circos plots showing structural variants and copy number alterations across the genome from all cases with WGS. Supplementary Figure S7. Chromothripsis assessment by targeted NGS (MSK-IMPACT) versus WGS. Supplementary Figure S8. RNAseq for non-recurrently amplified (KRAS, ERBB3, KDM5A) or deleted (TGFBR2, ARID1A) genes on chromothriptic chromosomes and TERT. Supplementary Figure S9. Comparison of chromothripsis characteristics in aSCLC vs other major lung cancer types. Supplementary Figure S10. Chromothripsis architecture in all cases profiled by whole-genome sequencing. Supplementary Figure S11. Chromothripsis in multi-sample analysis and genomic alterations in samples with histotype heterogeneity. Supplementary Figure S12. Cell cycle and p53 pathway deregulation in aSCLC. Supplementary Figure S13. Expression of potential therapeutic markers in aSCLC. [file cd-24-0286_supplementary_figures_s1-s13_suppsf1.pdf]

List of supplementary figures:

Supplementary Figure S1. Detailed morphologic and immunohistochemical findings: Case A01.

Supplementary Figure S2. Detailed morphologic and immunohistochemical findings: Case A08.

Supplementary Figure S3. Detailed morphologic and immunohistochemical findings: Case A17.

Supplementary Figure S4. Detailed morphologic and immunohistochemical findings: Case A20.

Supplementary Figure S5. Mutational signatures in atypical SCLC (aSCLC), never-smoker SCLC with *RB1*$^{-}$/*TP53*$^{-}$ (nsSCLC) and smoking-associated SCLC (sSCLC) analyzed by MSK-IMPACT.

Supplementary Figure S6. Circos plots showing structural variants and copy number alterations across the genome from all cases with WGS.

Supplementary Figure S7. Chromothripsis assessment by targeted NGS (MSK-IMPACT) versus WGS.

Supplementary Figure S8. RNAseq for non-recurrently amplified (*KRAS*, *ERBB3*, *KDM5A*) or deleted (*TGFBR2*, *ARID1A*) genes on chromothriptic chromosomes and TERT.

Supplementary Figure S9. Comparison of chromothripsis characteristics in aSCLC vs other major lung cancer types.

Supplementary Figure S10. Chromothripsis architecture in all cases profiled by whole-genome sequencing.

Supplementary Figure S11. Chromothripsis in multi-sample analysis and genomic alterations in samples with histotype heterogeneity.

Supplementary Figure S12. Cell cycle and p53 pathway deregulation in aSCLC.

Supplementary Figure S13. Expression of potential therapeutic markers in aSCLC.

Supplementary Figure S1

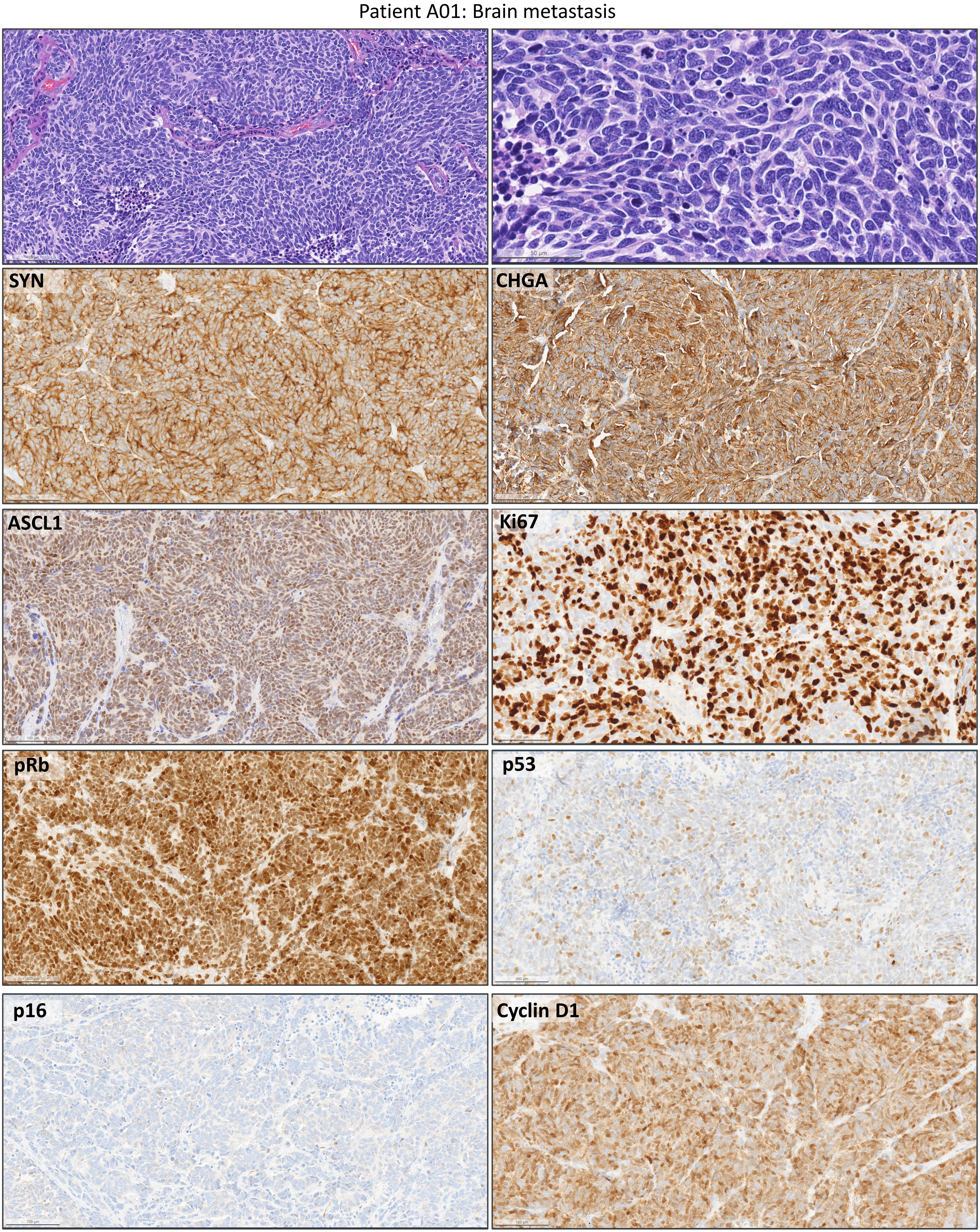

Supplementary Figure S1. Detailed morphologic and immunohistochemical findings: Case A01. Brain metastasis. H&E images illustrate classic morphologic features of small cell carcinoma, including crowded cells with high nuclear to cytoplasmic ratios such that cytoplasm is imperceptible. Cells show dense crowding and molding with abundant mitotic figures (>50/2 mm^2) and numerous apoptotic bodies. Areas of confluent necrosis are present (seen in lower-power H&E panel; top left). pRb shows retained expression, and p53 shows labeling in scattered cells, consistent with a wild-type pattern. Cyclin D1 overexpression corresponds to *CCND1* amplification associated with chromothripsis on chromosome 11.

Abbreviations: SYN synaptophysin, CHGA chromogranin A

Supplementary Figure S2

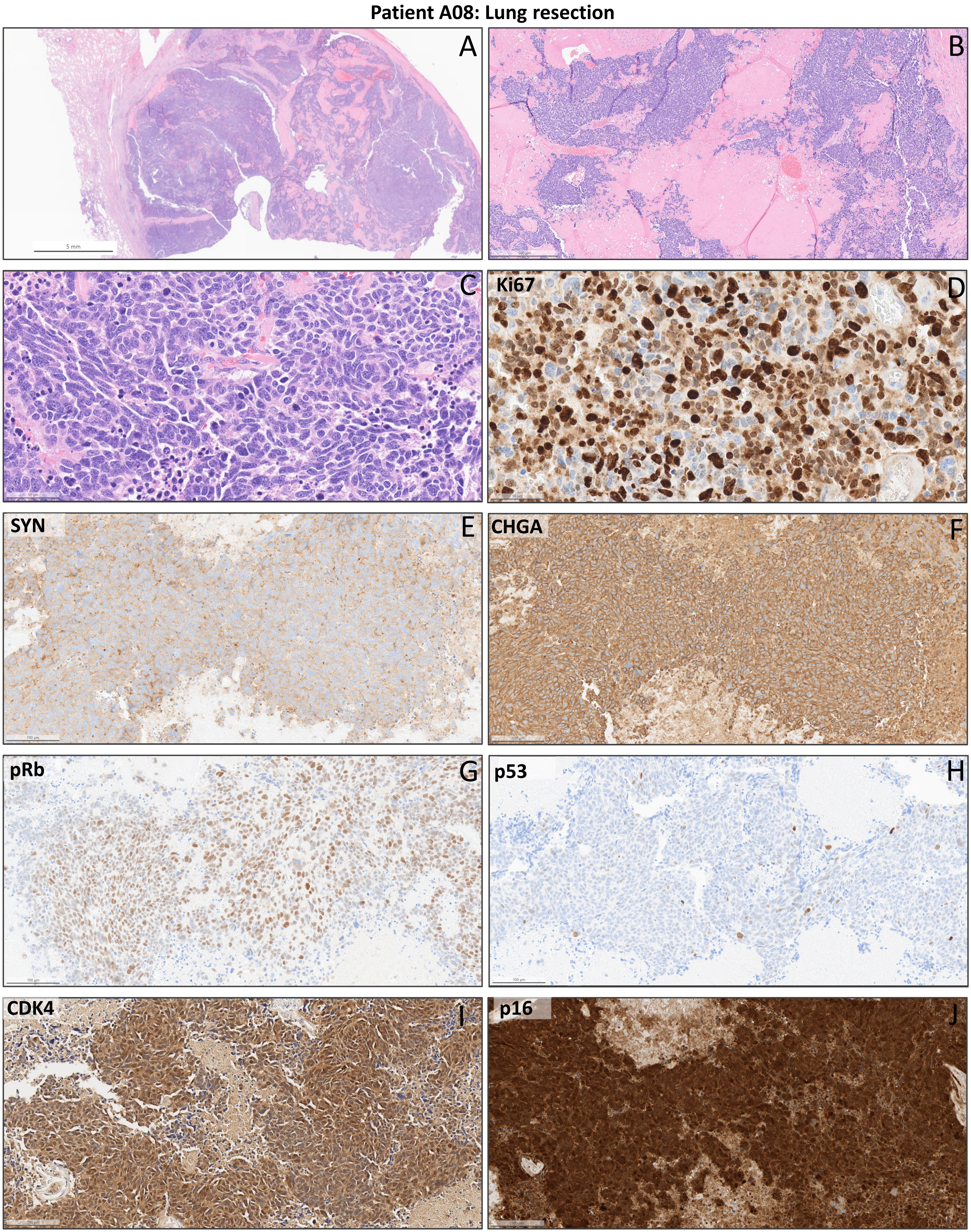

Supplementary Figure S2. Detailed morphologic and immunohistochemical findings: Case A08. Lung resection. H&E images illustrate classic morphology of small cell carcinoma, including extensive geographic/confluent necrosis, which is best seen low and intermediate magnification (panels A and B). At higher magnification (C), the tumor exhibits crowding, molding, and high nuclear to cytoplasmic ratios, abundant mitotic figures (~40/2 mm^2) and numerous apoptotic bodies. Ki67 (D), Synaptophysin (E), Chromogranin A (F) are shown. pRb (G) shows retained expression (lack of labeling localizes to non-viable cells surrounding areas of necrosis). p53 (H) shows labeling in scattered cells, consistent with a wild-type pattern. CDK4 (I) overexpression corresponds to *CDK4* amplification associated with chromothripsis on chromosome 12 (along with overexpression of Cyclin D2 and MDM2 – no shown). Overexpression of p16 (J) is paradoxical given that *RB1* is expressed and wild type (see Results).

Abbreviations: SYN synaptophysin, CHGA chromogranin A

Supplementary Figure S3

Patient A17: Lung tumor resection

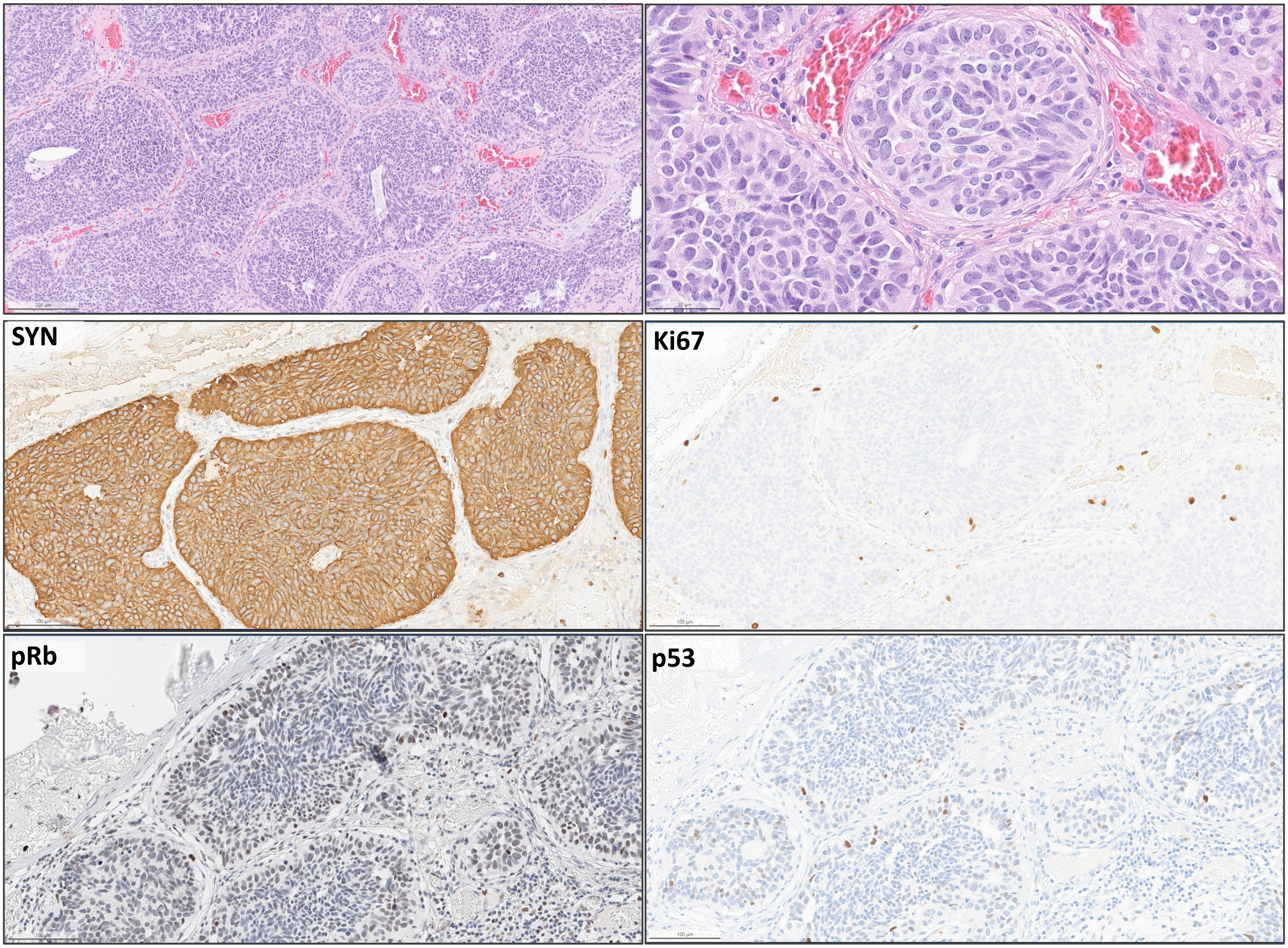

Patient A17: Neck lymph node metastasis excision

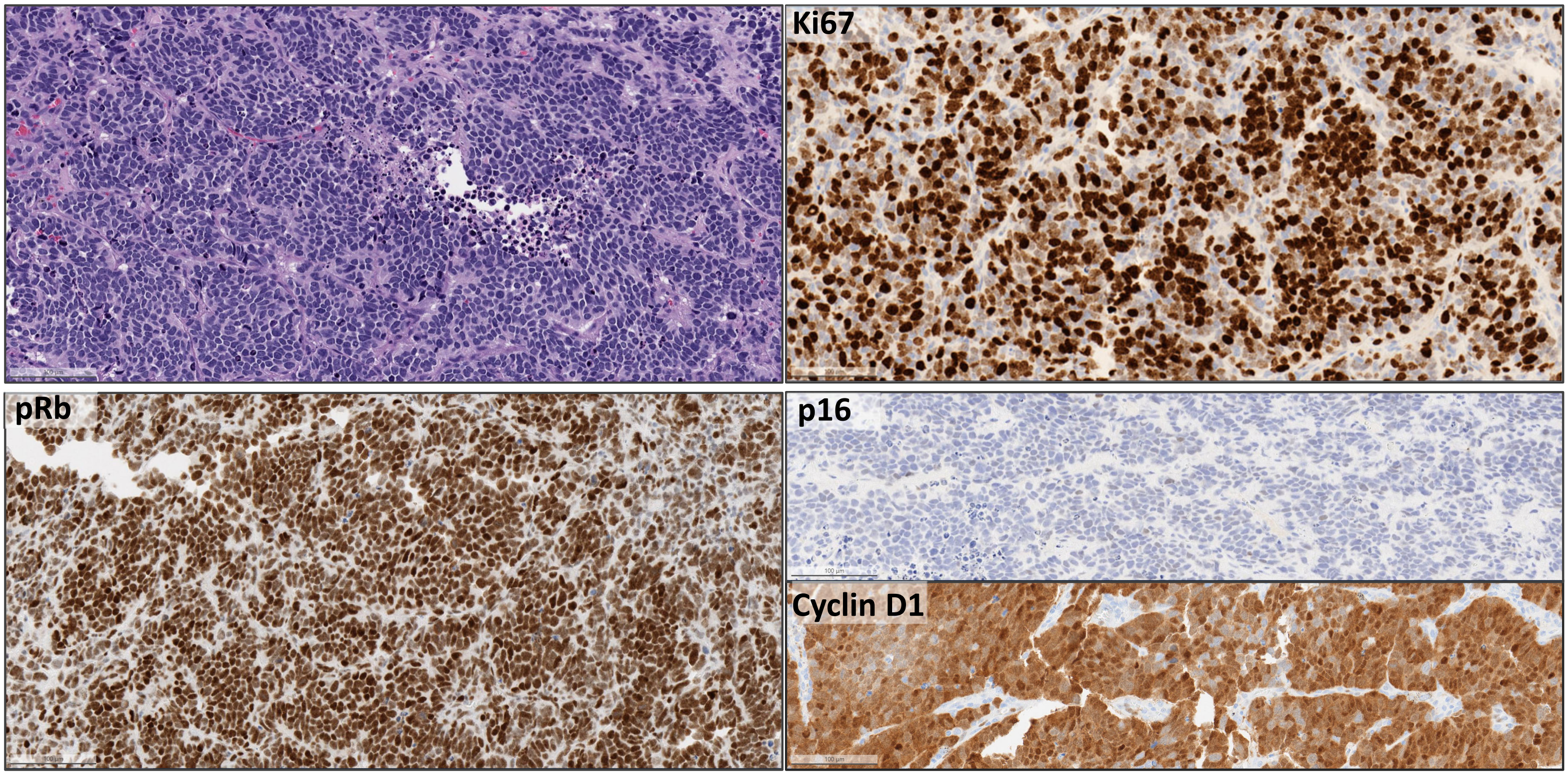

Supplementary Figure S3. Detailed morphologic and immunohistochemical findings: Case A17. Upper panel – Lung tumor resection; Lower panel - Neck lymph node metastasis excision. **Upper panel** shows classic histology of carcinoid tumor, composed of nests/islands of tumors cells with only focal necrosis and rare mitotic figures present. There were no areas of overt small cell carcinoma in this resection. pRb shows retained expression, and p53 shows labeling in scattered cells, consistent with a wild-type pattern. **Lower panel** (Neck lymph node metastasis excision; 5.3 years after primary tumor resection) shows dense proliferation of cells with high nuclear/cytoplasmic ratio, molding, extensive necrosis, and abundant mitotic figures and apoptotic bodies. Marked escalation of Ki67 proliferation index is illustrated. tNGS confirmed that the tumors are clonally related based on shared somatic genomic alterations (see **Supplementary Figure S11C**). pRb and p53 (not shown) retain a wild-type pattern; p16^{low}/Cyclin D1^{high} support pRb-proficiency. In this case, Cyclin D1 over-expression is associated with putative *CCND1* enhancer hijacking due to chromothripsis-mediated rearrangement.

Abbreviations: SYN synaptophysin, CHGA chromogranin A

Supplementary Figure S4A

Patient A20: Lung tumor resection

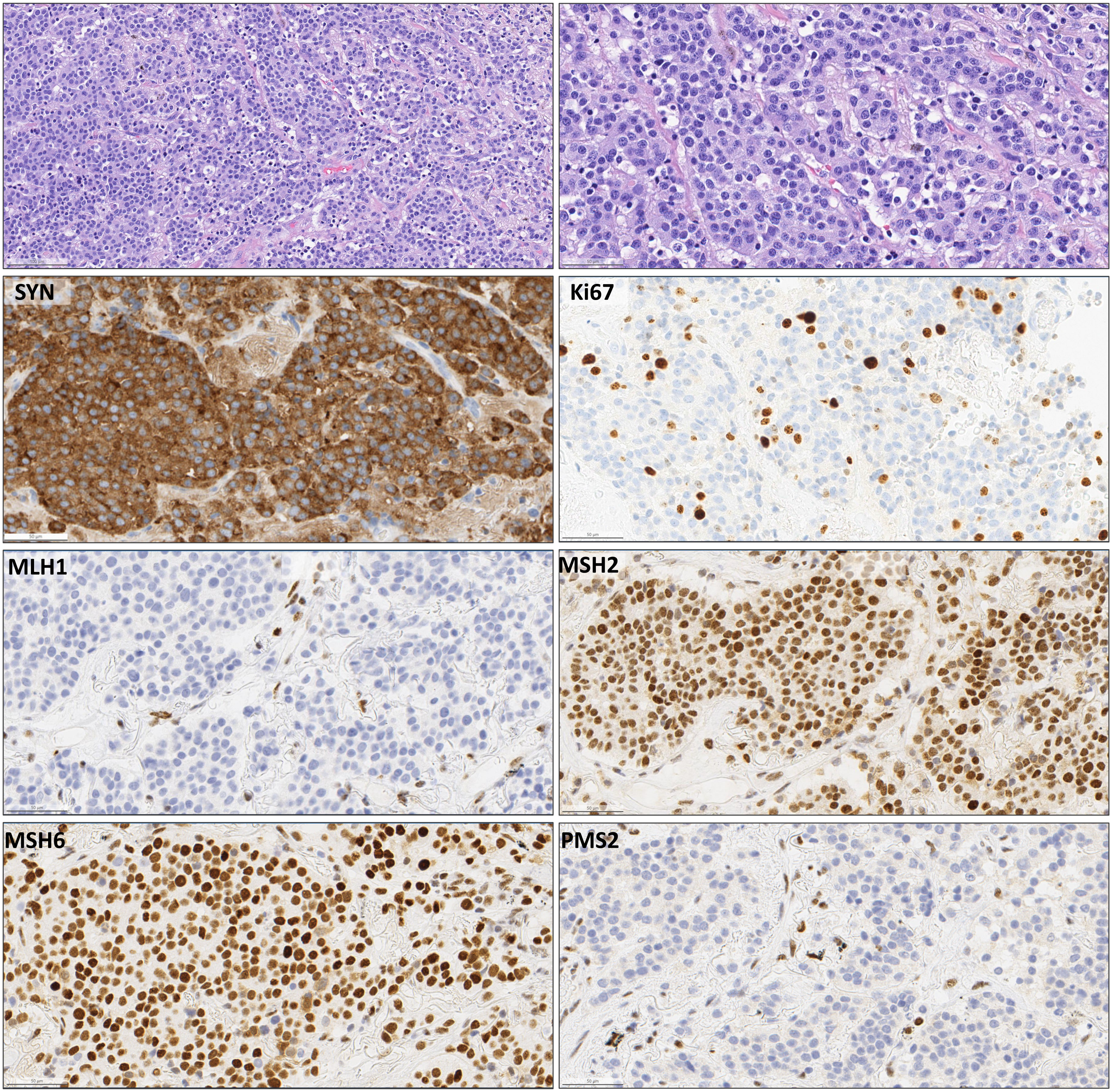

Supplementary Figure S4B

Patient A20: Biopsy of rib metastasis

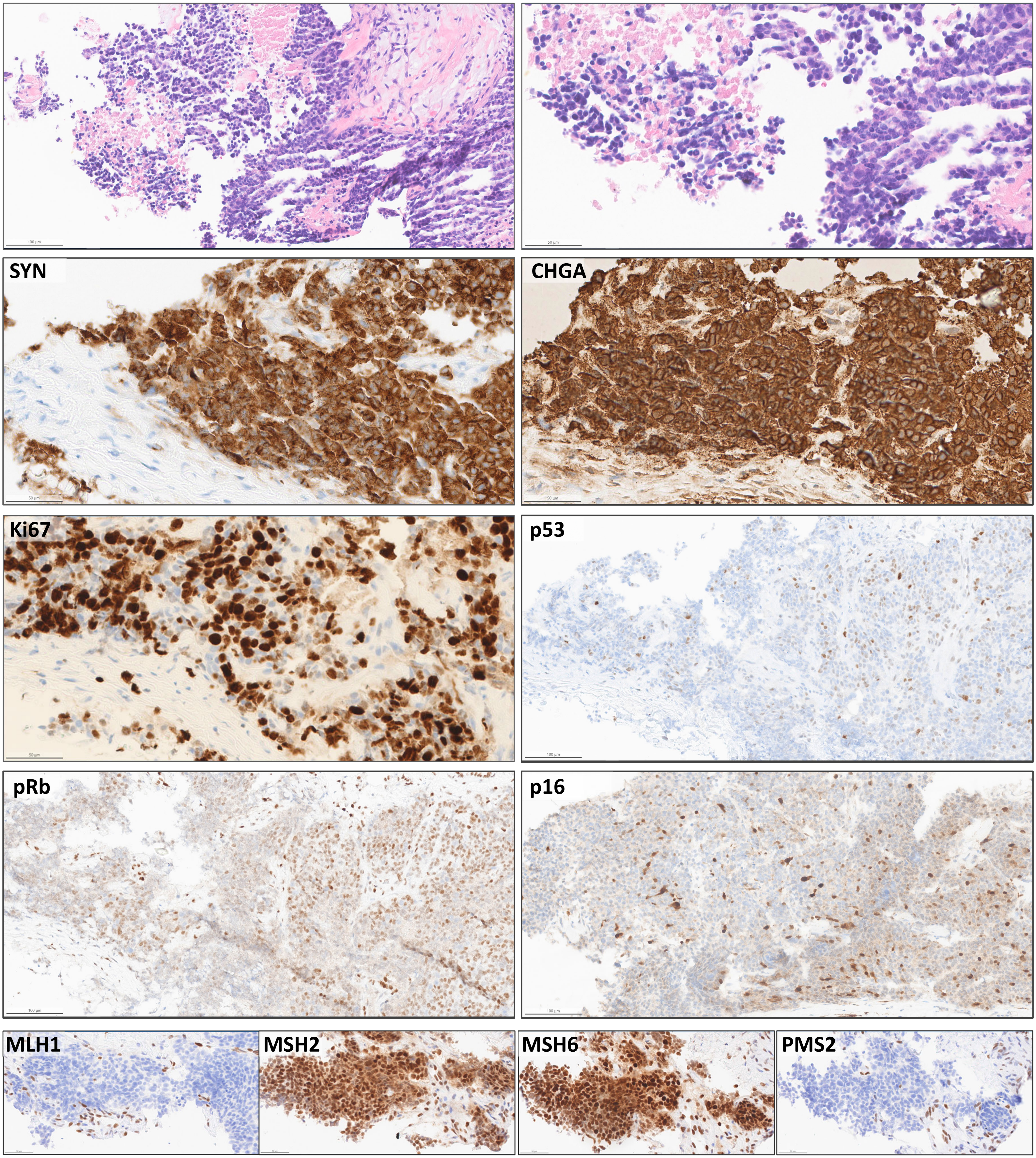

Supplementary Figure S4A and S4B. Detailed morphologic and immunohistochemical findings:
Case A20. Illustration of the sole case in the study associated with microsatellite instability (MSI). **S4A** (lung tumor resection) shows nested tumor with uniform, well-differentiated cells exhibiting low nuclear/cytoplasmic ratio such that eosinophilic cytoplasm is readily visible. There are rare mitotic figures and focal necrosis. No overt areas of small cell carcinoma were identified. Analysis of MSI-associate mismatch repair proteins confirms the loss of MLH1/PMS2, in line with somatic homozygous deletion of *MLH1*. **S4B**: Biopsy of rib metastasis, which was obtained 3.4 year after lung tumor resection, shows extensively necrotic tumor with crowded/molding cells with high nuclear/cytoplasmic ratios, and marked escalation of Ki67 proliferation rate. p53 shows labeling in scattered cells, consistent with a wild-type pattern, and pRb shows retained expression associated with p16 ^{low} profile.

Abbreviations: SYN synaptophysin, CHGA chromogranin A

Supplementary Figure S5

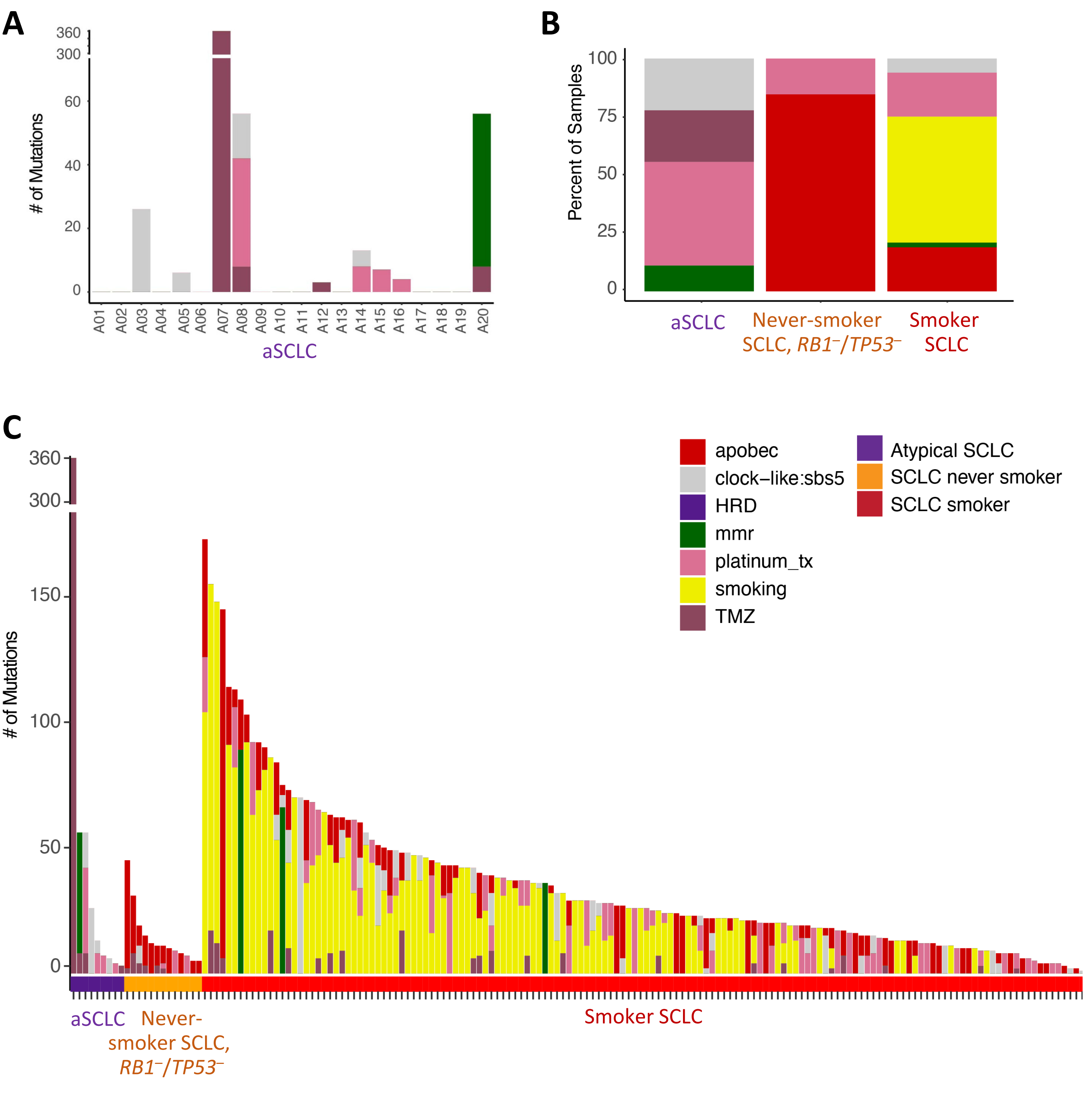

Supplementary Figure S5. Mutational signatures in atypical SCLC (aSCLC), never-smoker SCLC with *RB1⁻/TP53⁻* (nsSCLC) and smoking-associated SCLC (sSCLC) analyzed by MSK-IMPACT. Signatures were called in samples with >=5 SNVs. **A,** The number of mutations attributed to signatures passing FDR thresholds in aSCLC. **B,** Dominant mutational signature proportions across different classes of SCLC. **C,** Mutations attributed to signatures across all sample classes. Y-axis is split due to one aSCLC sample having a very large number of TMZ-associated mutations. Samples without a matched normal or <5 SNVs were culled from analysis. Total number of samples included and number evaluable for signature (>=5 SNVs and matched with normal) were as follows: aSCLC: 20 total, 16 evaluable, nsSCLC: 18 total; 17 evaluable; sSCLC: 187 total; 185 evaluable.

Supplementary Figure S6

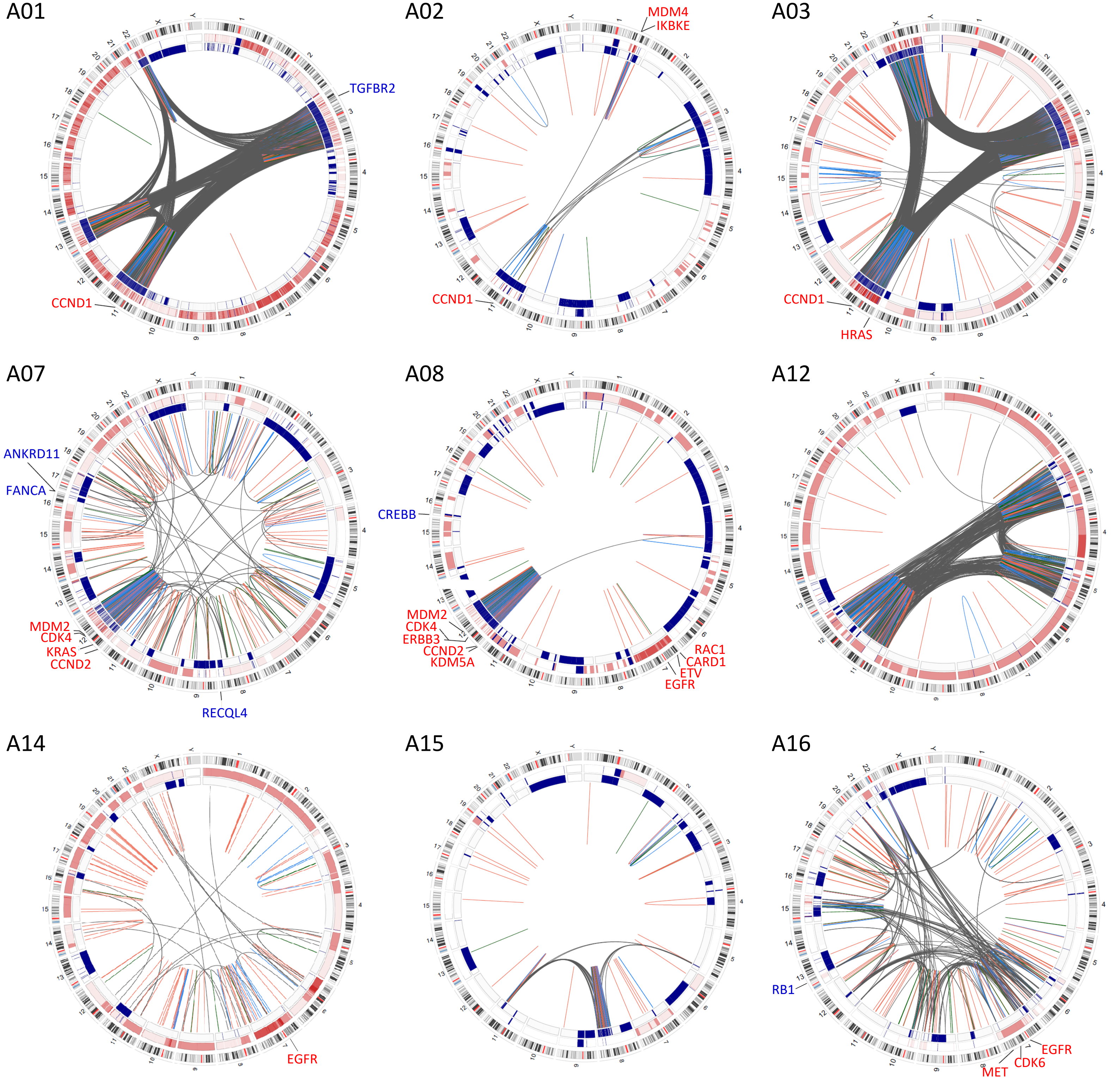

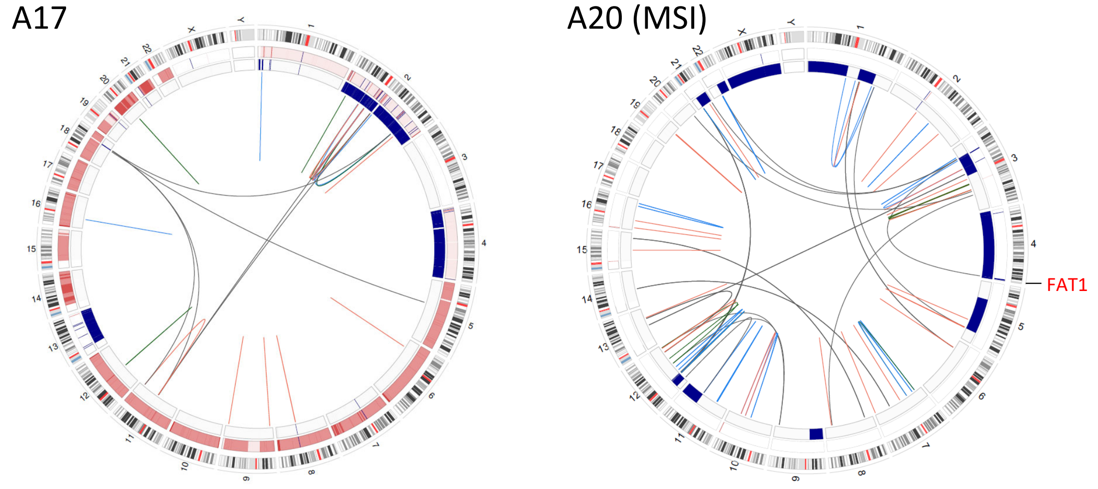

Supplementary Figure S6. Circos plots showing structural variants and copy number alterations across the genome from all cases with WGS. Gene amplifications (red) and deletions (blue) regarded as oncogenic or likely oncogenic by OncoKB are shown. See Figure 3A legend for further details.

Supplementary Figure S7

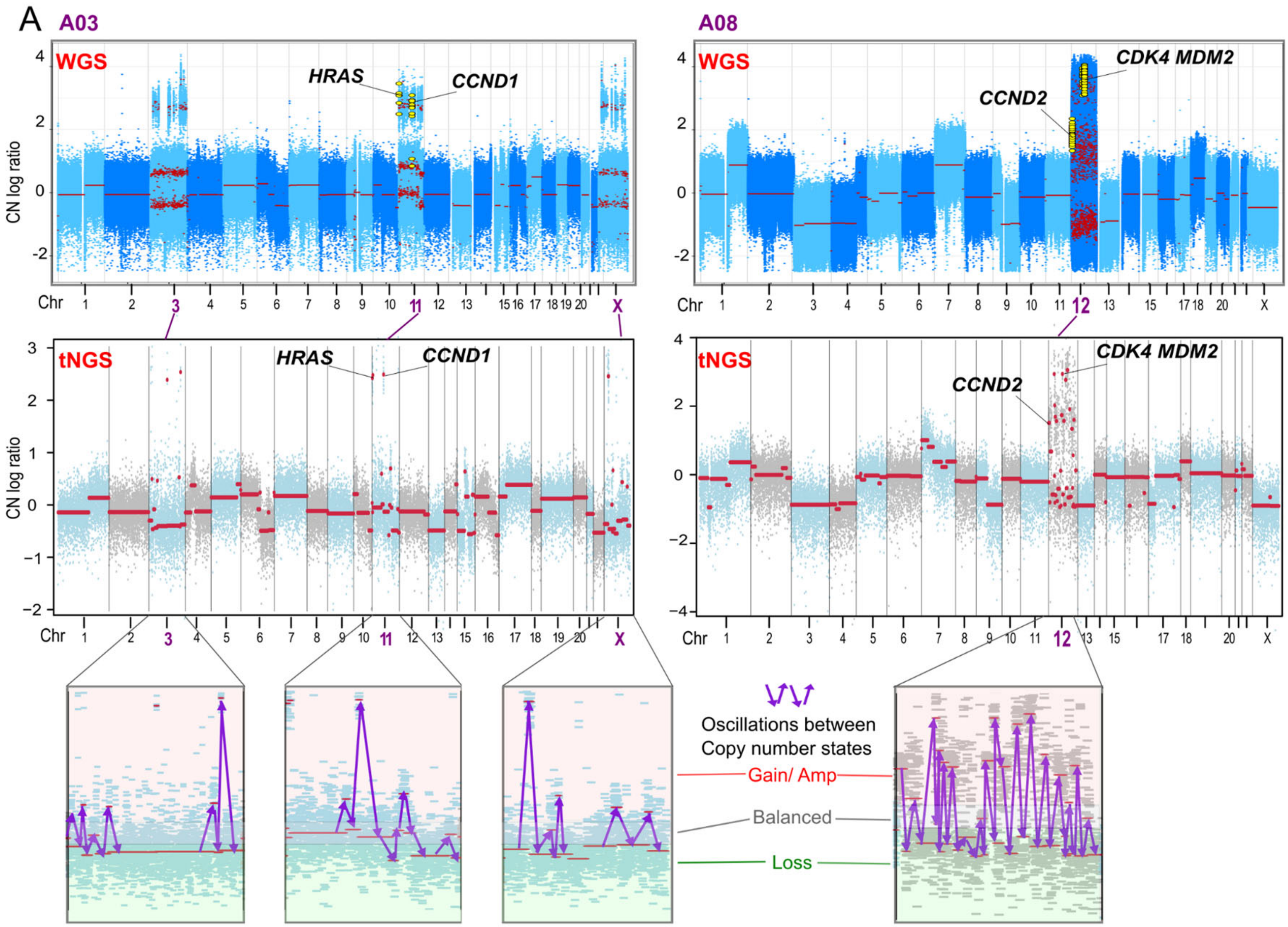

B

Chromothriptic Chromosome

Case ID	A01	A02	A03	A12	A07	A08	A14	A15	A16	A17	A20
1		V									
2										O	
3	V	O	V	V							
5				V							
6									V		
7									V		
8							O	O			
9									V		
11	V	O	V	V							
12				V	V	V					
13	V										
22	V										
X			V								

high-density (HD) chromothripsis by WGS
low-density (LD) chromothripsis by WGS
V detected by tNGS
O not detected by tNGS

C

tNGS vs. WGS by individual chromosomes

Groups		WGS: ChrT present	WGS: ChrT absent	Total
tNGS	ChrT present	17	0	17
	ChrT absent	5	231	236
Total		22	231	253

Sensitivity: 77%
Specificity: 100%

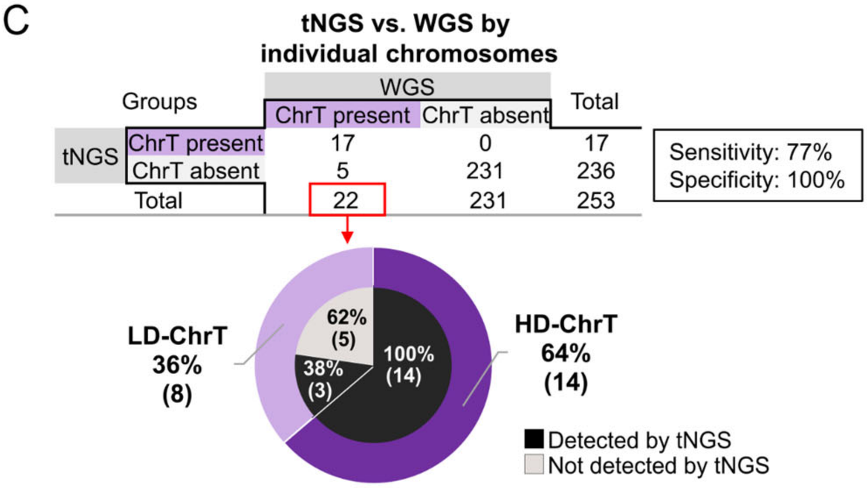

tNGS vs. WGS by case

Groups		WGS: ChrT present	WGS: ChrT absent	Total
tNGS	ChrT present	7	0	7
	ChrT absent	3	1	4
Total		10	1	11

Sensitivity: 70%
Specificity: 100%

tNGS: targeted NGS, WGS: whole genome sequencing
ChrT: chromothripsis, HD: high-density, LD: low density

Supplementary Figure S7. Chromothripsis assessment by targeted NGS (MSK-IMPACT) versus WGS. A, Copy number log-ratio plots of FACETS output from cases A03 (left) and A08 (right) illustrating the comparison of segmentation patterns by WGS (top) and tNGS (bottom). Key oncogene amplifications and chromothripsis of their source chromosomes were detected by both WGS and tNGS. Oscillations between copy number states (diploid or gain -> loss -> diploid or gain -> loss, etc) are readily identifiable by tNGS, although with lower density compared to WGS. **B,** Pairwise comparison of chromothripsis calls detected by WGS (gold standard) and tNGS showing that tNGS detected chromothripsis in most cases but missed single chromothripsis calls for chromosome 8 (2 cases), 2 (1 case), 3 (1 case), and 11 (1 case). Chromothripsis density was calculated as the total SVs in chromothripsis regions (respectively, as defined by Shatterseek) divided by region size (MB). Regions >=1 SV/MB were called high density and regions <1 SV/MB were called low density. All missed calls were low-density chromothripsis by WGS. **C,** Contingency tables evaluating the test performance of tNGS to detect chromothripsis using WGS as the gold standard. Comparison by individual chromosomes (top) and comparison by case (bottom) are shown. A pie chart (middle) showing the distribution of missed cases according to chromothripsis density indicates that most missed chromothripsis calls (5/8, 62%) involved low-density chromothripsis. Therefore, the main limitation of tNGS for chromothripsis detection is likely secondary to the low breadth of coverage of the targeted approach.

Supplementary Figure S8

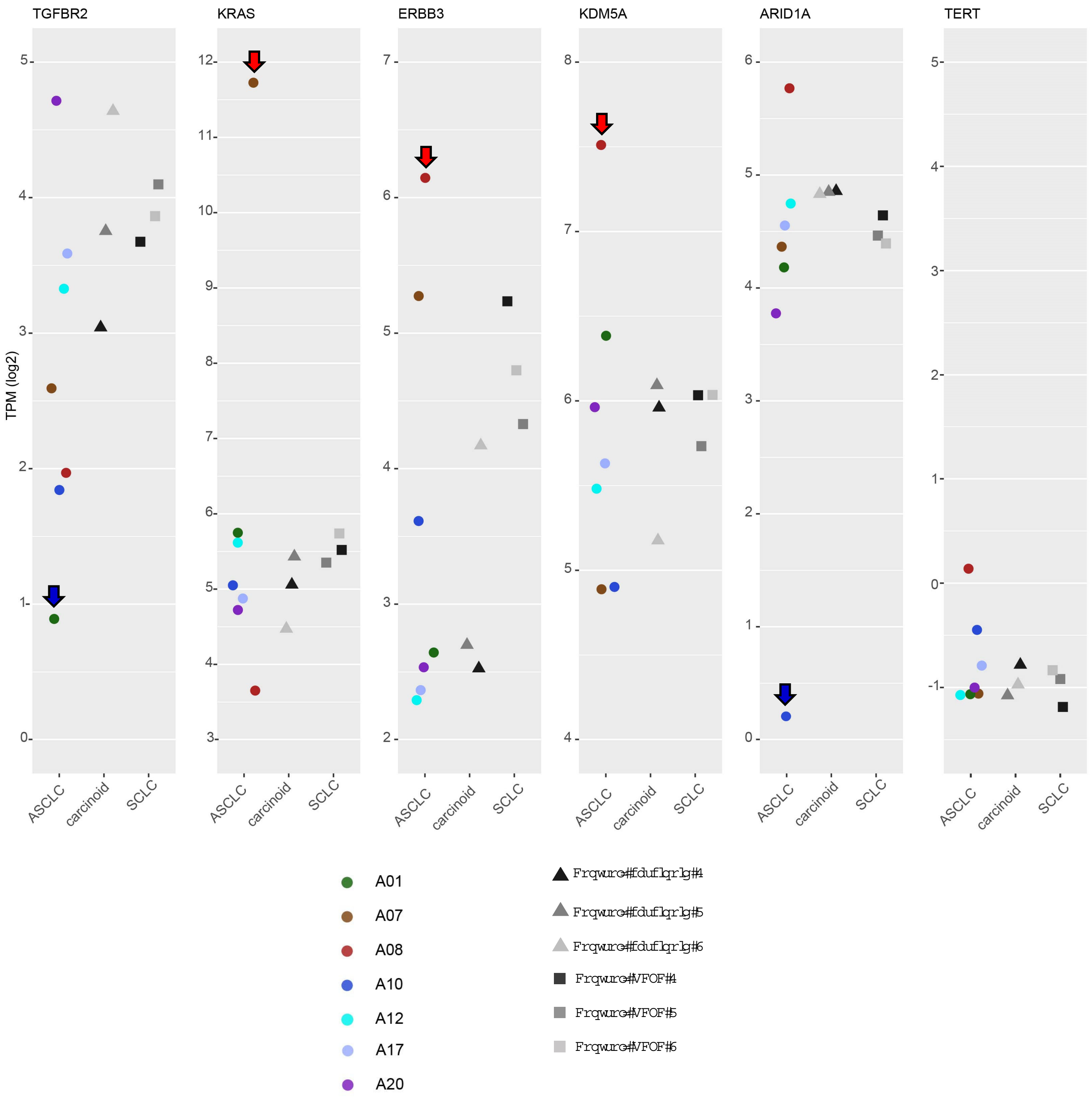

Supplementary Figure S8. RNAseq for non-recurrently amplified (*KRAS*, *ERBB3*, *KDM5A*) or deleted (*TGFBR2*, *ARID1A*) genes on chromothriptic chromosomes and TERT. Red arrows indicate cases with corresponding gene amplification on chromothriptic chromosomes, blue arrows indicate cases with corresponding gene deletions. TPM – transcripts per million

Supplementary Figure S9

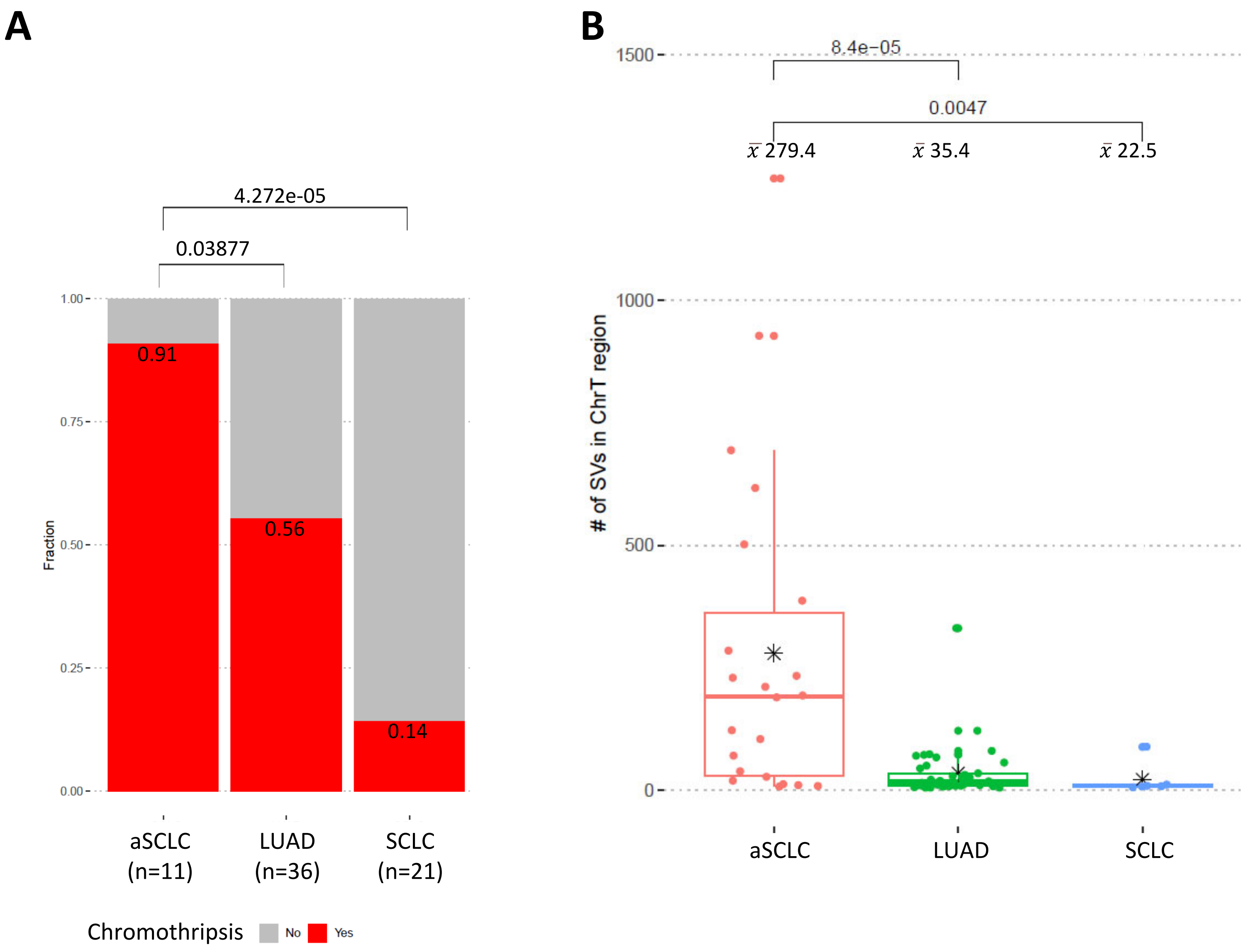

Supplementary Figure S9. Comparison of chromothripsis characteristics in aSCLC vs other major lung cancer types. A, Fraction of WGS samples with a chromothripsis events. Lung adenocarcinoma (LUAD) and *RB1*⁻/*TP53*⁻ SCLC WGS are from previous studies [26168399, 32025003], respectively. **B,** Total number of SVs per chromothripsis region defined by Shatterseek. The number of chromothripsis regions for aSCLC, LUAD and SCLC was 22, 41, and 6, respectively.

Supplementary Figure S10

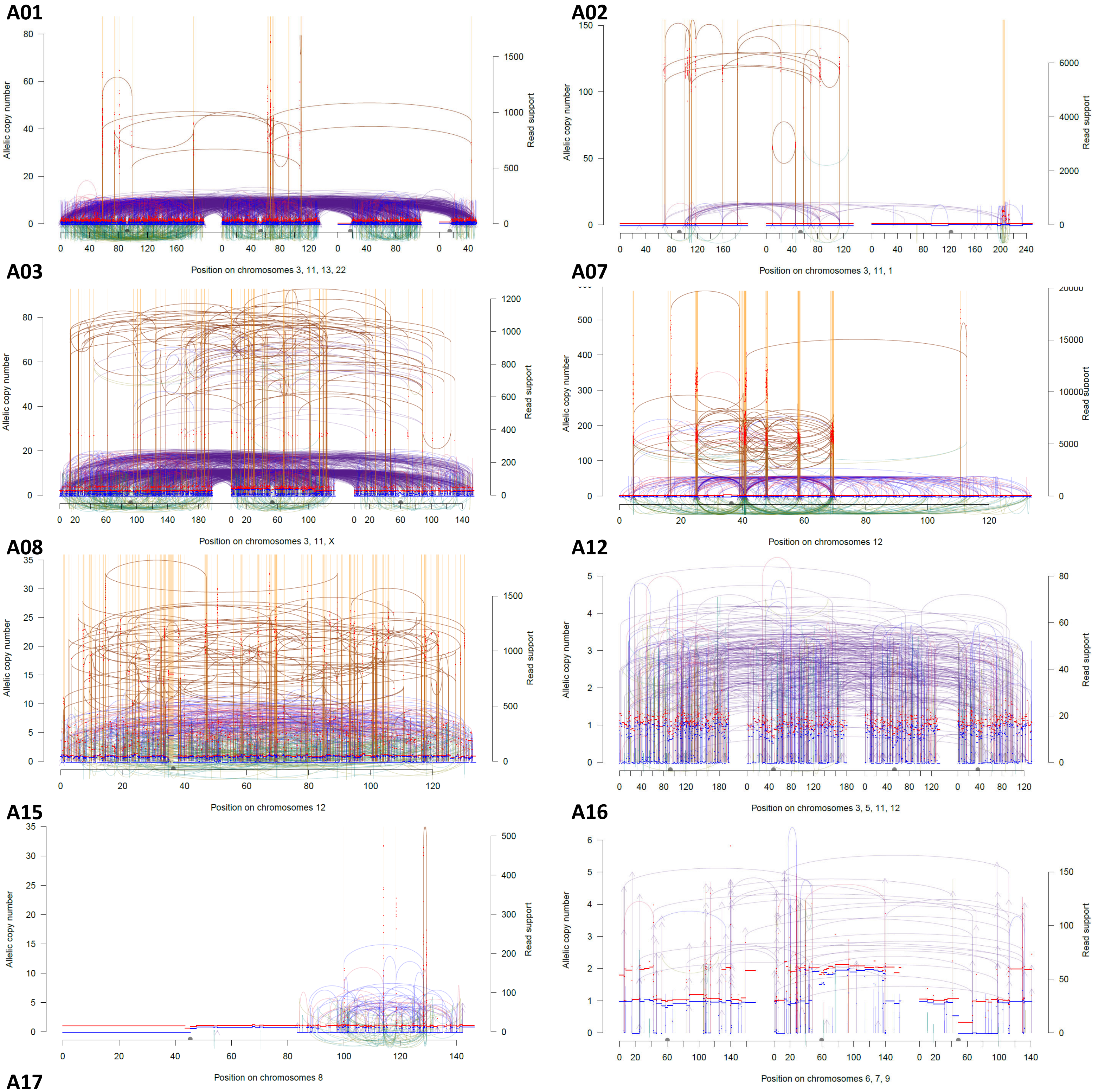

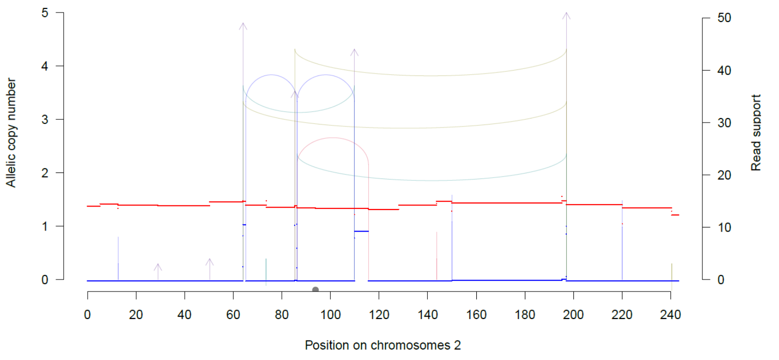

	A01	A02	A03	A07	A08	A12	A15	A16	A17
Full chromosome chromothripsis	3, 11, 13, 22	3	3, 11, X	12	12	3, 5, 11, 12	N	6,7,9	N
Partial chromosome chromothripsis		1,11					8		2
ecDNA	Y	Y	Y	Y	Y	N	N	N	N
Micro-nucleation	Y	N	Y	Y	Y	Y	N	N	N

Supplementary Figure S10. Chromothripsis architecture in all cases profiled by whole-genome sequencing. Integrated analysis of SVs and CNAs displaying signatures of chromothripsis. Only samples with high confidence chromothripsis calls are shown. See main Figure 4A for annotation. Summative table shows detected individual parameters for each case.

Supplementary Figure S11

A

A17 (multi-sample WGS)

Primary lung tumor (carcinoid histotype) — **Neck metastasis** (SCLC histotype)

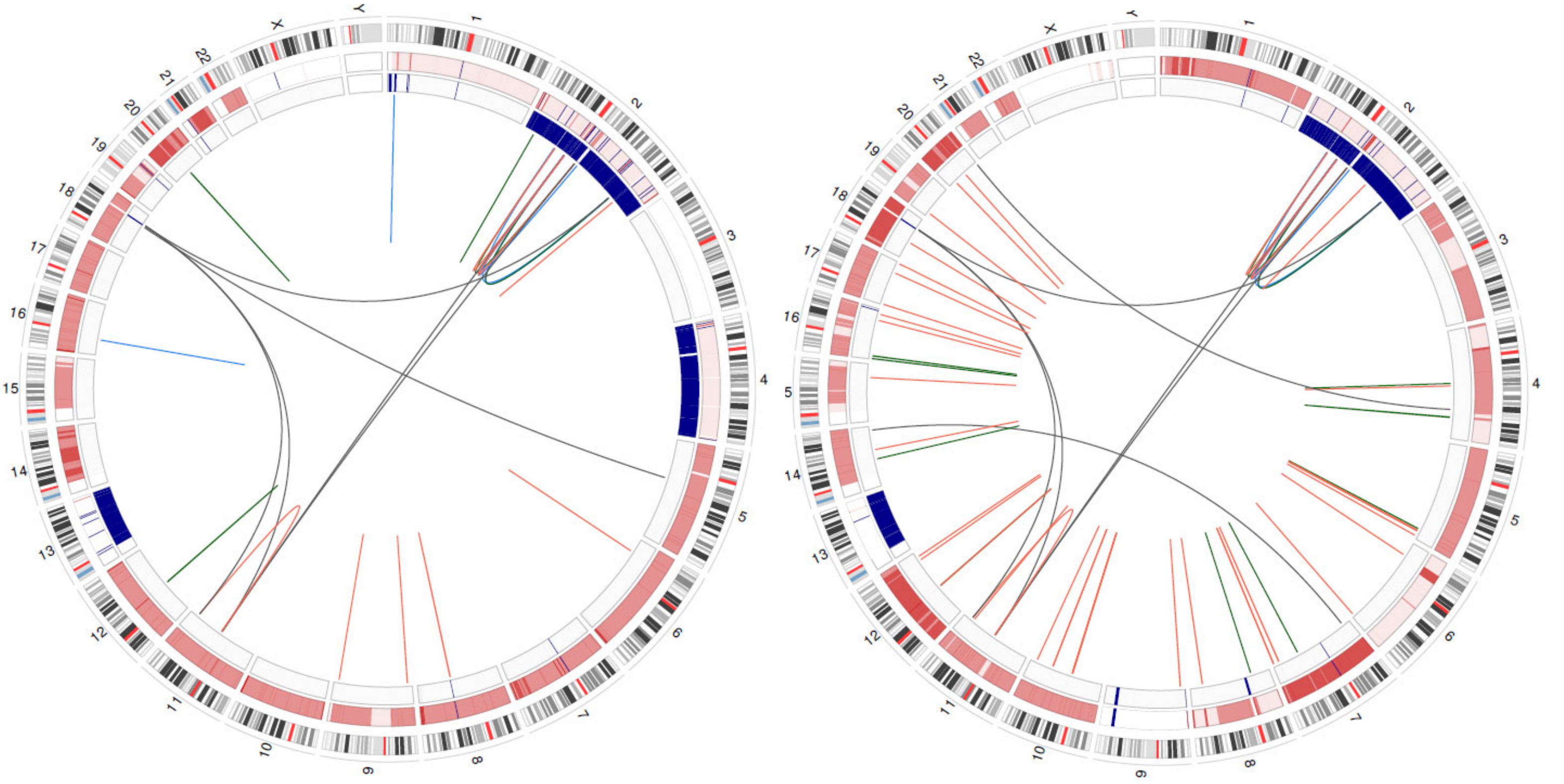

B

A08 (multi-sample targeted NGS)

Primary lung tumor — **Brain metastasis**

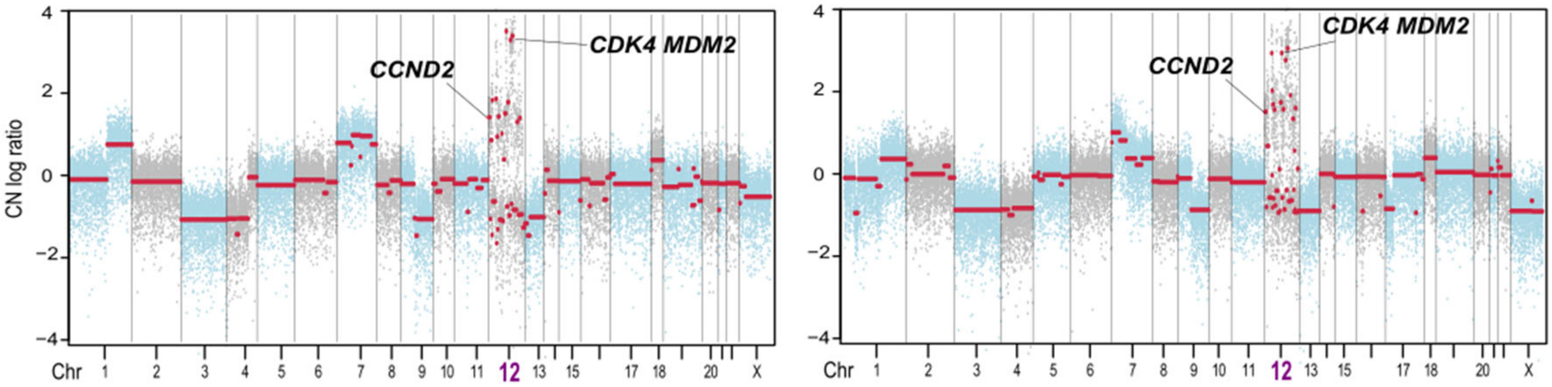

C

	A05		A17	
	Carcinoid (Sample 3)	SCLC (Sample 2)	Carcinoid (Sample 1)	SCLC (Sample 5)
Genomic alterations by MSK-IMPACT	- CCND1/FGF19/FGF4 amplification<br>- FGF3 (NM_005247 - 11q13.3) Deletion<br>- ATM exon49 p.A2415P (c.7243G>C)<br>- MEN1 exon10 p.K501Gfs*30 (c.1501_1514delAAGAAGCCA GCACT)	- CCND1/FGF19/FGF4 amplification<br>- ATM exon49 p.A2415P (c.7243G>C)<br>- MEN1 exon10 p.K501Gfs*30 (c.1501_1514delAAGAAGCCA GCACT)	- SMARCA4 exon26 p.R1192C (c.3574C>T)<br>- U2AF1 exon6 p.R156H (c.467G>A)	- PRDM1 exon5 p.P300L (c.899C>T)<br>- SMARCA4 exon24 p.F1102L (c.3304T>C)<br>- U2AF1 exon6 p.R156H (c.467G>A)
TMB	1.8	1.8	1.8	2.6
Coverage	635X	636X	407X	623X

Supplementary Figure S11. Chromothripsis in multi-sample analysis and genomic alterations in samples with histotype heterogeneity. A, B, Circos plots (A17: two samples profiled by WGS, top) and copy number log-ratio plot (A08: two samples profiled by tNGS, bottom) showing significant overlap in the patterns of chromothripsis and copy number changes between primary and metastatic tumors. The findings support the concept that chromothripsis is conserved event in the evolution of atypical SCLC. **C,** Genomic alterations by MSK-IMPACT in cases with histotype heterogeneity (SCLC and carcinoid) in different samples.

Supplementary Figure S12

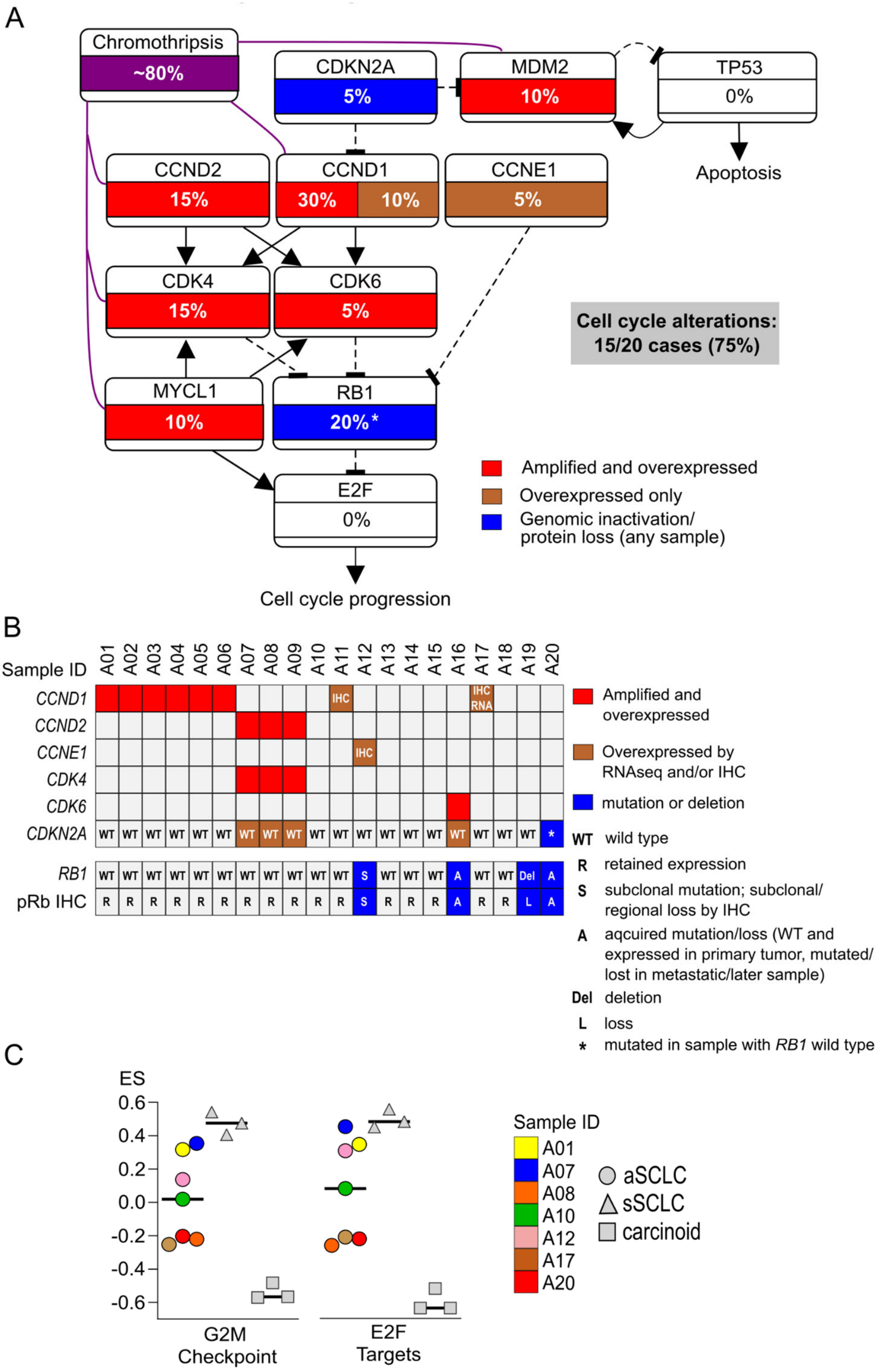

Supplementary Figure S12. Cell cycle and p53 pathway deregulation in aSCLC. A, Summative diagram for cell cycle and p53 pathway deregulation based on integrated DNA sequencing, RNAseq and IHC data. In patients with multiple samples, percentages reflect alteration detected in any tested sample. *All *RB1* alterations/loss were acquired (A16, A20) or subclonal (A12) or involved metastatic sample in the absence of primary tumor sample analysis and with low p16 (A19), interpreted as likely acquired *RB1* mutation (see **Supplementary Table S1)**. **B,** Corresponding Oncoprint depicting alterations in cell cycle genes/proteins in individual patients. **C,** Gene pathway analysis by RNAseq showing upregulation of pro-proliferative signatures in atypical SCLC relative to carcinoids, in some cases to the level seen in conventional SCLC.

Supplementary Figure S13

A

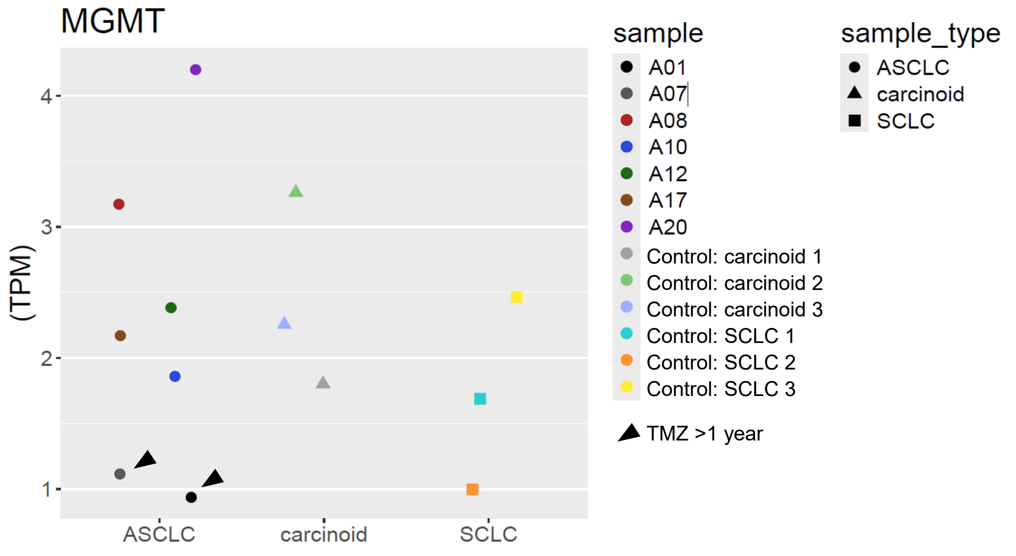

B

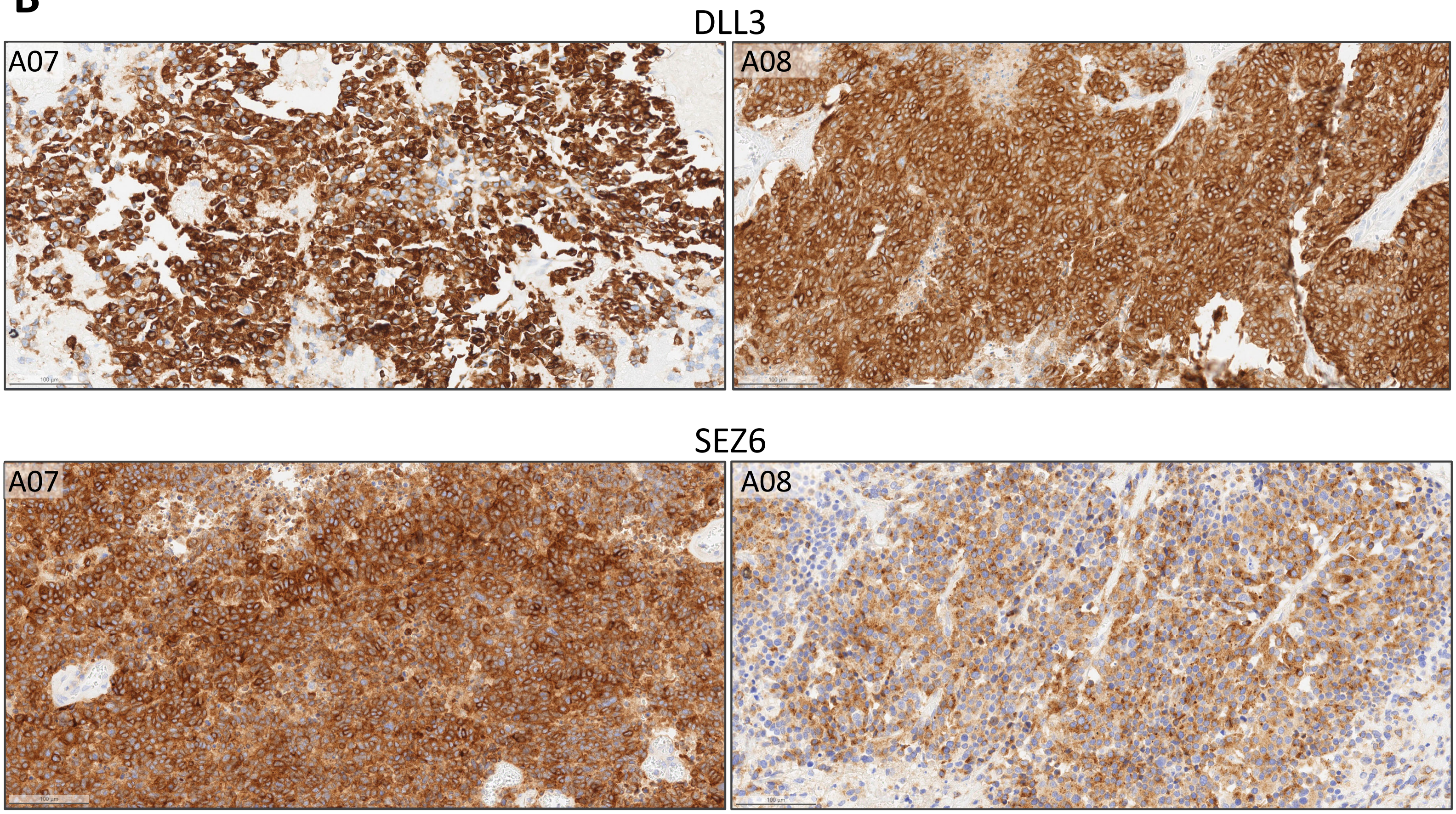

Supplementary Figure S13. Expression of potential therapeutic markers in aSCLC. A, RNAseq for of O^6-methylguanine-DNA methyltransferase MGMT. B. Expression of DLL3 and SEZ6 in representative cases of aSCLC by IHC.
